# Estimates of bladder cancer burden attributable to high fasting plasma glucose: Findings of the Global Burden of Disease Study 2019

**DOI:** 10.1002/cam4.6219

**Published:** 2023-06-23

**Authors:** Ying Wu, Yujiao Deng, Zhijun Dai, Yubo Ma, Lijuan Lyu, Chen Lei, Yi Zheng, Yizhen Li, Ziming Wang, Jie Gao

**Affiliations:** ^1^ Department of Urology The Second Affiliated Hospital of Xi'an Jiaotong University Xi'an China; ^2^ Department of Nephrology The Second Affiliated Hospital of Xi'an Jiaotong University Xi'an China; ^3^ Department of Breast Surgery The First Affiliated Hospital, College of Medicine, Zhejiang University Hangzhou China; ^4^ Department of Oncology The Second Affiliated Hospital of Xi'an Jiaotong University Xi'an China; ^5^ Department of Endocrinology The General Hospital of Ningxia Medical University Yinchuan China

**Keywords:** bladder cancer, DALY, death, high fasting plasma glucose

## Abstract

**Background:**

High fasting plasma glucose (FPG) has been listed as one of the risk factors for bladder cancer. We here estimated the global, regional, and national levels of bladder cancer burden attributable to high FPG from 1990 to 2019.

**Methods:**

Bladder cancer data attributable to high FPG were extracted from the Global Burden of Disease Study 2019, and analyzed by age, sex, year, and location. Age‐standardized rates were utilized to evaluate the burden between different populations. The temporal trend of the burden was estimated through the Joinpoint analysis.

**Results:**

In 2019, high FPG contributed to 22,823.33 (95% uncertainty interval [UI], 4694.88–48,962.26) deaths and 399,654.91 (95% UI, 81,609.35–865,890.95) disability‐adjusted life years (DALYs) of bladder cancer globally. Since 1990, the global age‐standardized death and DALY rates of bladder cancer attributable to high FPG increased apparently by 39.18% and 41.48%, respectively. During the last 30 years, high FPG‐related age‐standardized death and DALY rates of bladder cancer have increased in all countries. In 2019, Central Europe showed the greatest high FPG‐related age‐standardized death and DALY rates of bladder cancer, but Andean Latin America had the lowest rates. Nationally, Lebanon showed the greatest high FPG‐related age‐standardized death and DALY rates of bladder cancer in 2019. High FPG‐attributable deaths and DALYs of bladder cancer were more considerable among males and older people. Countries with high SDI showed higher levels of age‐standardized death and DALY rates of bladder cancer due to high FPG and presented remarkable upward trends in rates in the last 30 years.

**Conclusions:**

Globally, the high FPG‐associated bladder cancer burden has remarkably increased in all countries, and showed a higher level among countries with higher SDI. Monitoring FPG levels among patients with bladder cancer is critical to lower the corresponding burden.

## 1 INTRODUCTION

Bladder cancer has been the 12th frequently diagnosed cancer and the 13th leading cause of cancer death worldwide, with 573 thousand incident cases and 213 thousand deaths in 2020.^1^ Various risk factors and medical conditions were associated with the initiation and progression of bladder cancer, such as smoking, high fasting plasma glucose (FPG), diabetes condition, occupational exposures, and dietary factors.^2^, ^3^

High FPG was reported to be associated with 6.50 million deaths and 172.07 million disability‐adjusted life years (DALYs) of all causes around the world in 2019.^4^ Meanwhile, high FPG has been demonstrated to be not only a well‐known risk factor for some cardiovascular diseases but also an independent risk factor for various cancers.^5^ Owing to urbanization and changing lifestyles, high FPG has become more common and has increased worldwide, especially among people in higher‐income countries.^6^ Prediabetes and diabetes conditions were the common manifestations of high FPG, causing an increasing burden worldwide and becoming a great challenge for human health. Growing evidence indicated that high FPG, prediabetes, and diabetes conditions were related to the increased risk of various cancers.^7^, ^8^, ^9^, ^10^ Among which, bladder cancer risk was observed to rise with the duration of diabetes.^7^ Similarly, compared with non‐diabetics, bladder cancer patients with diabetes presented an elevated risk for cancer‐specific mortality, progression, and recurrence of the disease.^8^ Thus, the findings of prior studies suggested that elevated FPG and diabetes conditions have become a threat to patients with cancers.

Previous studies have reported the burden of total cancer attributable to high FPG, but there is no specific and detailed analysis of corresponding bladder cancer burden.^11^ However, understanding the global, regional, and national level and trends in bladder cancer burden due to high FPG is essential to better allocate the resources and take measures to reduce the corresponding burden. Thus, we here estimated the spatiotemporal trend of bladder cancer attributable to high FPG worldwide from 1990 to 2019 based on the most up‐to‐date data of GBD 2019, providing supported data for the monitoring and management of FPG levels in bladder cancer patients worldwide.

## 2 METHODS

### 2.1 Overview

Bladder cancer and its disease burden attributable to high FPG was obtained from the GBD Study, which updated and provided the data of 369 diseases and corresponding risk factors biennially across the world. All countries and territories were categorized into five socio‐demographic index (SDI) quintiles (high, high‐middle, middle, low‐middle, low SDI), and divided into 21 regions by the geographic vicinity and epidemiological resemblance. The SDI was assessed according to average educational attainment, fertility rate, and composite assessment of the economic growth of different countries.

### 2.2 Estimation of high FPG attributed burden

High FPG, an ongoing exposure measured in units of mmol/L, is determined as the population's mean FPG and computed by taking the person‐year weighted average of the FPG levels linked to the lowest risk of death in pooled assessments of prospective cohort research. It is defined as any concentration above the theoretical minimum risk exposure level (TMREL) (4.8–5.4 mmol/L). This criterion includes any high FPG conditions including diabetes (diagnosed at 7 mmol/L). Thus, a systematic review has been performed regarding high FPG and diabetes in the previous GBD study.^5^ The FPG exposure and relative risk (RR) models incorporated data from 549 different sources. The mean FPG was utilized for exposure estimations when both the mean FPG level and diabetes prevalence were reported, or it was evaluated through the prevalence of diabetes via an ensemble distribution from the GBD studies. The RR of FPG on bladder cancer was derived from dose–response meta‐analyses based on prospective cohort studies. Modeling the mean FPG over location, year, age, and sex level was conducted through a spatiotemporal Gaussian process regression framework.^5^ The population‐attributable fraction (PAF) is the percentage of risk that would be decreased in a specific year if historical exposure to high FPG levels were reduced to the desired exposure level. Attributing deaths and DALY of bladder cancer to high FPG were calculated using PAF for age, sex, location, and year.

### 2.3 Statistical analyses

The burden of bladder cancer resulting from high FPG was estimated according to age, sex, year, and location. All estimates of this study were reported with their 95% uncertainty intervals (UIs). This uncertainty for every estimate may be caused by various data sources, modeling uncertainty, potential error in data processing, and data manipulation. It was determined as the percentile range of the distribution from the 2.5th to the 97.5th in 1000 draw levels. Age‐standardized rates (ASRs) were utilized to compare the burden between different populations to reduce the influence of population structures. As previously reported, temporal patterns of bladder cancer burden attributable to high FPG were analyzed through the Joinpoint Regression Model to identify best‐fitting points through Joinpoint software (version 4.7.0) from the US National Cancer Institute.^12^ The annual percentage changes (APCs) were calculated and reported with 95% confidence intervals (CIs) to indicate the direction of the temporal trend (a negative value indicated a downward trend, while a positive value represented an upward trend). R studio software (R Core Team, version 4.1) was utilized to analyze the data. A significant level was defined as a *p* less than 0.05.

## 3 RESULTS

### 3.1 Global estimates

In 2019, there were 228,735 (95% UI, 210,743–243,193) deaths and 4,392,583 (95% UI, 4,090,438–4,702,733) DALYs of bladder cancer documented globally. In GBD studies, smoking and high FPG were two primary risk factors identified for bladder cancer. In 2019, smoking contributed to 33.89% (95% UI, 25.88–41.80) deaths and 36.76% (95% UI, 28.46–44.00) DALYs of all bladder cancer, whereas high FPG was responsible for 9.98% (95% UI, 2.09%–21.3%) deaths and 9.11% (95% UI, 1.87%–19.59%) DALYs of total bladder cancer. Although smoking resulted in more deaths and DALYs of bladder cancer, its attributable bladder cancer burden decreased apparently since 1990, with significant declines by −29.3% (95% UI, −35.02% to −22.63%) and −30.03% (95% UI, −36.16% to −22.82%) in global age‐standardized death and DALY rates, respectively (Figure S1). In contrast to it, high FPG contributed to an increase in the global burden of bladder cancer over the last 30 years, with remarkable increases in age‐standardized death and DALY rates by 39.18% (95% UI, 34.45%–48.53%) and 41.48% (95% UI, 36.69%–50.44%), respectively (Table 1).

**TABLE 1:** Bladder cancer burden attributable to high FPG.

Categories	Death			DALY		
	Counts (in thousand) (95% UI)	ASR per 100,000 population (95% UI)	Percentage change in ASR, 1990–2019 (%) (95% UI)	Counts (in thousand) (95% UI)	ASR per 100,000 population (95% UI)	Percentage change in ASR, 1990–2019 (%) (95% UI)
Global	22.82 (4.69–48.96)	0.3 (0.06–0.64)	39.18 (34.45–48.53)	399.65 (81.61–865.89)	4.97 (1.02–10.74)	41.48 (36.69–50.44)
Sex						
Female	5.53 (1.01–12.51)	0.13 (0.02–0.28)	33.46 (29.2–40.2)	89.97 (16.23–202.8)	2.05 (0.37–4.62)	36.66 (32–43.57)
Male	17.29 (2.99–38.6)	0.53 (0.09–1.18)	39.98 (35.15–47.91)	309.69 (53.41–693.92)	8.6 (1.49–19.14)	41.42 (36.57–49.46)
Socio‐demographic index						
High SDI	8.64 (1.79–18.46)	0.4 (0.08–0.86)	45.11 (38.41–57.9)	134.48 (28–290.46)	6.74 (1.4–14.55)	46.83 (40.39–58.82)
High‐middle SDI	6.72 (1.35–14.57)	0.33 (0.07–0.72)	36.53 (32.01–44.74)	117.33 (23.4–255)	5.73 (1.14–12.43)	38.89 (34.38–46.92)
Middle SDI	4.29 (0.86–9.28)	0.2 (0.04–0.43)	34.34 (27.7–43.83)	84.26 (16.93–181.77)	3.54 (0.71–7.61)	41.45 (33.33–52.33)
Low‐middle SDI	2.2 (0.46–4.7)	0.19 (0.04–0.4)	46.3 (40.05–56.31)	43.5 (9.02–93.92)	3.37 (0.7–7.26)	50.83 (44.37–61.27)
Low SDI	0.97 (0.2–2.16)	0.24 (0.05–0.53)	39.13 (32.44–49.95)	19.82 (4.09–44.39)	4.27 (0.88–9.57)	44.05 (37.06–55.21)
21 GBD regions						
Andean Latin America	0.08 (0.02–0.18)	0.15 (0.03–0.34)	54.91 (45.62–69.86)	1.32 (0.28–2.99)	2.45 (0.51–5.53)	60.84 (50.78–75.79)
Australasia	0.15 (0.03–0.33)	0.27 (0.05–0.58)	53.24 (41.84–71.13)	2.17 (0.41–4.74)	4.08 (0.77–8.91)	62.54 (51.32–81.3)
Caribbean	0.2 (0.04–0.43)	0.39 (0.08–0.83)	26.42 (20.49–36.12)	3.48 (0.75–7.43)	6.74 (1.46–14.37)	29.69 (23.44–39.74)
Central Asia	0.14 (0.03–0.31)	0.23 (0.05–0.52)	94.96 (84.42–114.35)	3.15 (0.63–7.03)	4.55 (0.91–10.1)	98.46 (87.56–117.84)
Central Europe	1.33 (0.27–2.92)	0.58 (0.12–1.28)	45.14 (39.03–55.61)	24.47 (4.93–53.98)	10.98 (2.21–24.25)	49.49 (43.07–60.09)
Central Latin America	0.46 (0.11–0.97)	0.21 (0.05–0.43)	10.26 (6.15–15.15)	8.45 (1.9–17.69)	3.68 (0.83–7.7)	15.85 (11.64–21.47)
Central sub‐Saharan Africa	0.12 (0.02–0.32)	0.33 (0.06–0.82)	25.56 (16.74–37.98)	2.64 (0.48–6.68)	5.84 (1.09–14.8)	29.57 (20.25–42)
East Asia	3.15 (0.6–6.95)	0.17 (0.03–0.38)	16.39 (3.58–28.89)	58.93 (11.21–130.6)	2.93 (0.56–6.49)	20.11 (6.48–33.01)
Eastern Europe	0.62 (0.12–1.42)	0.17 (0.03–0.4)	37.86 (33.29–44.48)	12.13 (2.33–27.78)	3.43 (0.66–7.86)	38.43 (33.99–45.18)
Eastern sub‐Saharan Africa	0.23 (0.05–0.52)	0.19 (0.04–0.42)	25.34 (17.01–35.95)	4.53 (0.95–10.42)	3.24 (0.68–7.47)	31.76 (22.58–44.24)
High‐income Asia Pacific	1.04 (0.2–2.35)	0.18 (0.03–0.4)	15.15 (11.55–19.93)	14.54 (2.79–32.81)	2.88 (0.56–6.52)	15.03 (11.34–20.12)
High‐income North America	3.48 (0.74–7.33)	0.51 (0.11–1.08)	53.97 (45.32–72.48)	56.4 (11.89–118.26)	8.66 (1.83–18.18)	52.67 (44.48–68.78)
North Africa and Middle East	1.68 (0.35–3.64)	0.46 (0.1–1)	74.11 (63.15–92.43)	35.7 (7.32–78.83)	8.84 (1.84–19.4)	82.63 (70.48–102.77)
Oceania	0.01 (0–0.03)	0.25 (0.06–0.56)	42.51 (33.86–59.75)	0.34 (0.08–0.77)	5.07 (1.16–11.32)	48.4 (39.53–64.82)
South Asia	2.23 (0.47–4.84)	0.19 (0.04–0.4)	48.25 (40.25–61.34)	45.29 (9.45–97.97)	3.39 (0.71–7.36)	51.87 (44.59–64.57)
Southeast Asia	0.86 (0.18–1.87)	0.18 (0.04–0.39)	52.33 (44.3–66.44)	15.73 (3.28–34.85)	2.91 (0.62–6.38)	56.48 (47.65–71.22)
Southern Latin America	0.32 (0.06–0.68)	0.37 (0.07–0.79)	68.09 (56.77–86.74)	5.53 (1.11–12)	6.51 (1.3–14.11)	69.63 (58.19–88.33)
Southern sub‐Saharan Africa	0.15 (0.03–0.32)	0.31 (0.07–0.67)	38.9 (32.12–50.82)	2.95 (0.67–6.46)	5.63 (1.28–12.17)	51.08 (42.7–65.25)
Tropical Latin America	0.56 (0.12–1.22)	0.25 (0.05–0.54)	12.3 (7.85–19.08)	9.77 (2.06–21.38)	4.15 (0.88–9.08)	13.21 (8.84–20.14)
Western Europe	5.74 (1.19–12.32)	0.53 (0.11–1.15)	44.07 (37.9–55.7)	86.95 (18.03–189.26)	8.91 (1.85–19.45)	45.23 (39.33–56.47)
Western sub‐Saharan Africa	0.27 (0.05–0.64)	0.2 (0.04–0.46)	43.85 (34.18–57.83)	5.19 (1.02–12.22)	3.28 (0.65–7.75)	48.45 (37.97–62.66)

Abbreviations: ASR, age‐standardized rate; DALY, disability‐adjusted life year; GBD, global burden of disease; UI, uncertainty interval.

**FIGURE 1:**
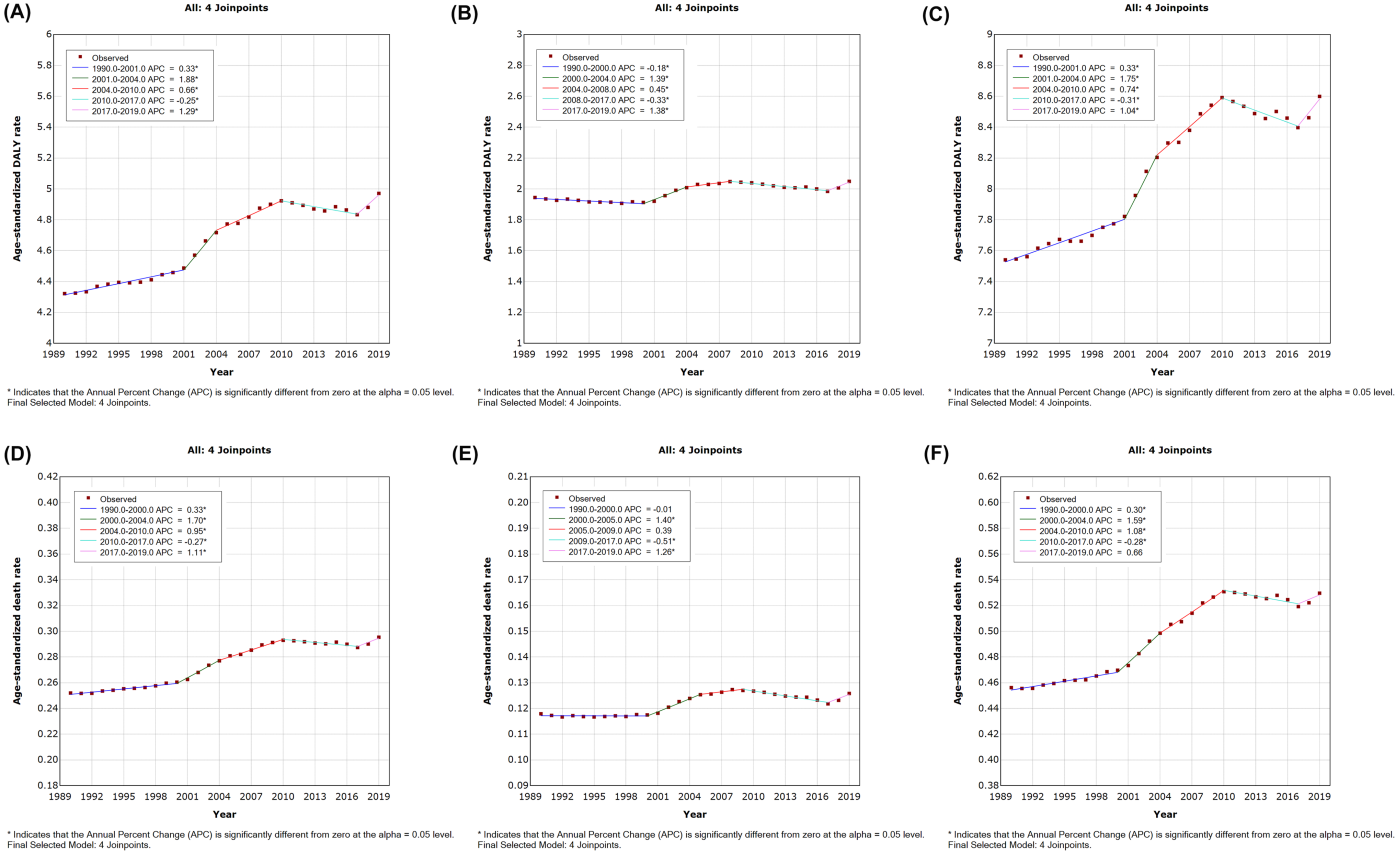
Global trends of bladder cancer burden attributable to high FPG from 1990 to 2019. (A) Age‐standardized DALY rate for both sexes; (B) age‐standardized DALY rate for female; (C) age‐standardized DALY rate for male; (D) age‐standardized death rate for both sexes; (E) age‐standardized death rate for female; (F) age‐standardized death rate for male. DALY, disability‐adjusted life year; FPG, fasting plasma glucose.

In 2019, 22,823.33 (95% UI, 4694.88–48,962.26) deaths of bladder cancer attributable to high FPG were recorded with an ASR of 0.30 (95% UI, 0.06–0.64) per 100,000 population. In the same year, 399,654.91 (95% UI, 81,609.35–865,890.95) DALYs of high FPG‐attributable bladder cancer were documented with an ASR of 4.97 (95% UI, 1.02–10.74) per 100,000 population (Table 1). As shown in Figure 1, global high FPG‐related age‐standardized death and DALY rates of bladder cancer have significantly increased since 1990, especially from 2001 to 2004 (age‐standardized DALY rate: APC = 1.9% [95% CI, 1.0%–2.7%], *p* < 0.05; age‐standardized death rate: APC = 1.7% [95% CI, 1.3%–2.1%], *p* < 0.05), whereas they decreased from 2010 to 2017 with the same APCs of −0.3% (95% CI, −0.4% to −0.1%) (*p* < 0.05) (Figure 1). For different sexes, the rises in age‐standardized death and DALY rates of bladder cancer due to high FPG were much more apparent in males than in females, although the declines in rates occurred earlier in females (Figure 1).

### 3.2 Regional estimates

**FIGURE 2:**
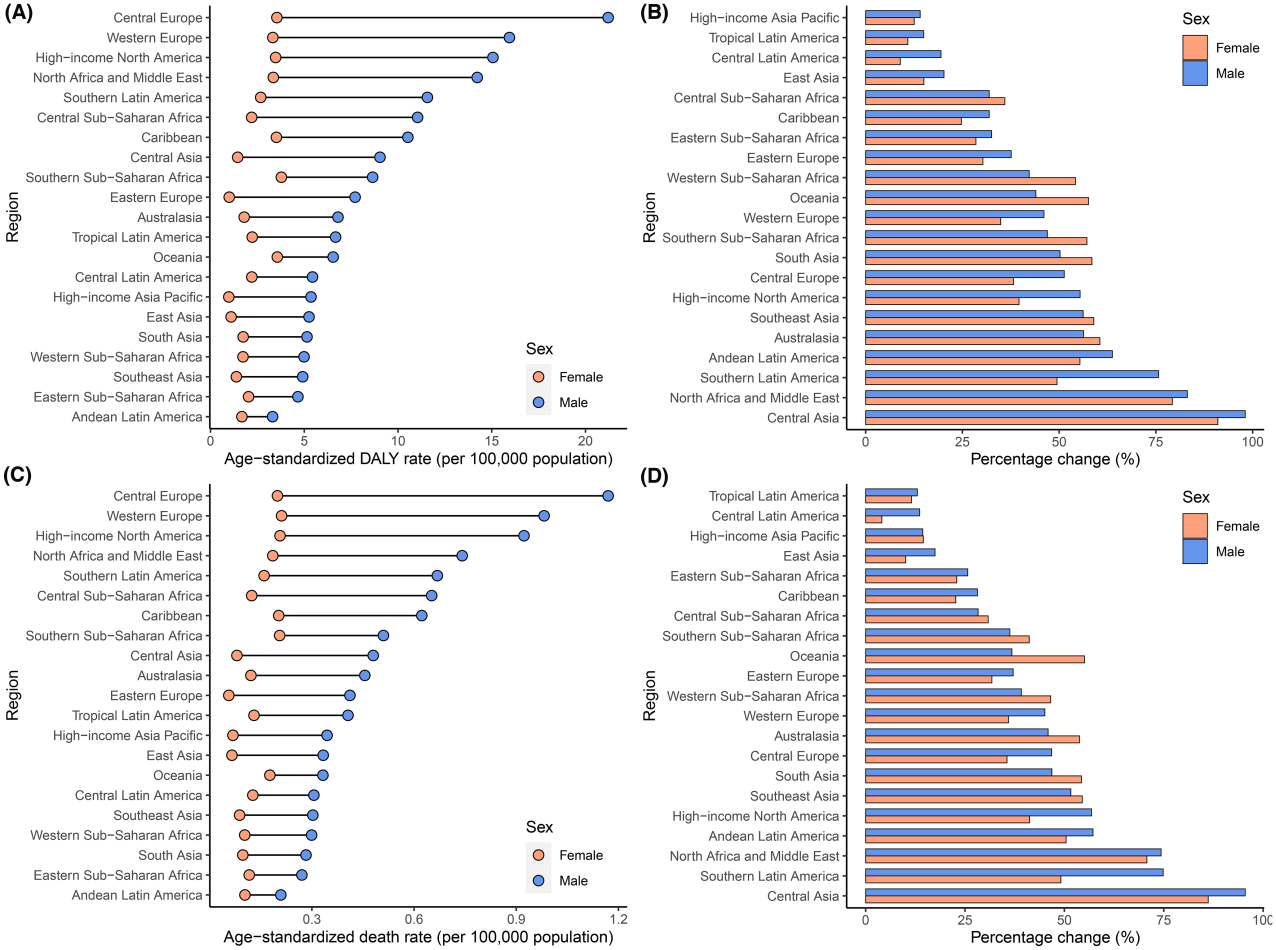
Regional age‐standardized rates (per 100,000 populations) of bladder cancer attributable to high FPG in 2019 and their percentage changes in rates for different sexes from 1990 to 2019. (A) Age‐standardized DALY rate in 2019; (B) percentage change in age‐standardized DALY rate, 1990–2019; (C) age‐standardized death rate in 2019; (D) percentage change in age‐standardized death rate, 1990–2019. DALYs, disability‐adjusted life years; FPG, fasting plasma glucose.

Regionally, in 2019, Central Europe, followed by Western Europe, showed the greatest age‐standardized DALY and death rates of bladder cancer due to high FPG. By contrast, Andean Latin America presented the lowest age‐standardized death and DALY rates of bladder cancer due to high FPG in the same year (Table 1). When it comes to different sexes, the same results can be found among males. However, for females, the high FPG‐attributed ASDR of bladder cancer was highest in Western Europe, but lowest in Eastern Europe in 2019. Regarding the age‐standardized DALY rate among females, it was greatest in Southern sub‐Saharan Africa but lowest in High‐income Asia Pacific (Figure 2).

Since 1990, high FPG‐associated age‐standardized death and DALY rates of bladder cancer have increased for both sexes in all regions, especially in Central Asia, North Africa and Middle East, and Southern Latin America. However, the slightest increases in age‐standardized death and DALY rates were observed in Central Latin America and Tropical Latin America, respectively (Table 1). For different sexes, high FPG‐associated age‐standardized death and DALY rates of bladder cancer increased the least in Central Latin America for females. However, the least increases for males were found in High‐income Asia Pacific for age‐standardized DALY rate, and in Tropical Latin America for ASDR (Figure 2).

### 3.3 National estimates

At the national level, Lebanon, Qatar, and Bahrain were the three countries with the greatest age‐standardized death and DALY rates of bladder cancer due to high FPG, whereas the rates were lowest in Mongolia for both sexes in 2019 (Table S1). Regarding different sexes, the same results can be found among males in the same year. However, for females, the lowest age‐standardized death and DALY rates of bladder cancer due to high FPG were also found in Mongolia, whereas the highest rates were observed in Qatar.

Over the last 30 years, all countries have seen increases in age‐standardized death and DALY rates of bladder cancer attributable to high FPG for both sexes, with the greatest increases in rates being observed in Luxembourg (Table S2). By contrast, Mexico showed the slightest increase in ASDR of bladder cancer due to high FPG, whereas Singapore had the lowest increment in age‐standardized DALY rate from 1990 to 2019. When it comes to differences among sexes, high FPG‐related age‐standardized death and DALY rates of bladder cancer presented the slightest increases in Ethiopia for males, but in Singapore for females (Table S2).

### 3.4 Age and sex patterns

**FIGURE 3:**
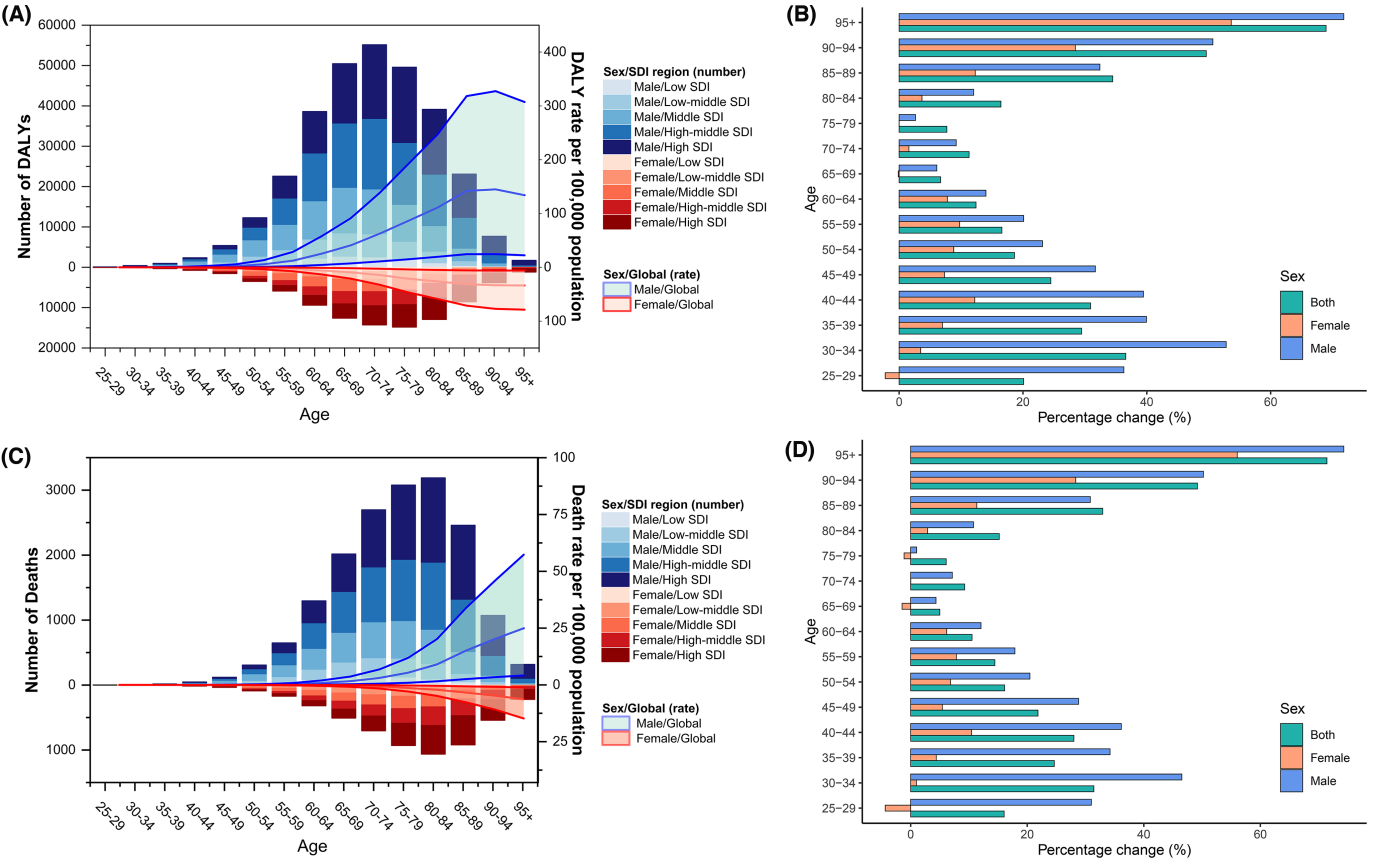
Bladder cancer burden attributable to high FPG among different sexes and age groups in 2019 and percentage changes in age‐specific rates (per 100,000 population) from 1990 to 2019. (A) DALYs (the bars represent the number of DALYs) and age‐specific DALY rates (the lines with 95% UI indicate DALY rate per 100,000 population); (B) percentage changes in age‐specific DALY rates from 1990 to 2019; (C) deaths (the bars represent the numbers of deaths) and age‐specific death rates (the lines with 95% UI indicate death rates per 100,000 population); (D) percentage changes in age‐specific death rates from 1990 to 2019. DALYs, disability‐adjusted life years; FPG, fasting plasma glucose; UI, uncertainty interval.

As shown in Figure 3, for different sexes, the bladder cancer deaths and DALYs attributable to high FPG was much more considerable among males compared with their female counterparts, with more than triple deaths and DALYs in 2019 (Table 1 and Figure 3). Similar results can be found in age‐specific death and DALY rates. Meanwhile, in all age groups, high FPG contributed to the most deaths and DALYs of bladder cancer among patients in countries with high SDI. For different age groups, high FPG‐attributable deaths of bladder cancer peaked among people aged 80–84 years for both sexes. However, DALYs of bladder cancer due to high FPG reached the highest levels among males aged 70–74 years and females aged 75–79 years (Figure 3). As Figure 3 illustrates, age‐specific death rates in both sexes increased with age with the greatest rates observed in the over 95 age group, but the age‐specific DALY rate reached the highest level in males aged 90–94 years and in females aged over 95 years. Through the past 30 years, high FPG contributed to the most deaths and DALYs of bladder cancer among people aged 75–79 from 1990 to 2019 (Figure S2). However, the age‐specific death and DALY rates of bladder cancer due to high FPG show obvious increases, with the largest increases in rates observed in the over 95 age group and lowest increases in rates found in 65–69 age group. For males, the greatest increases in high FPG‐associated age‐specific death and DALY rates of bladder cancer were found in males aged over 95, but the lowest increases in rates were seen in males aged 75–79 years. For females, the biggest increases in age‐specific death and DALY rates were also seen in females aged over 95, but apparent declines in rates were observed in females aged 25–29 years, 65–69 years, and 75–79 years. Interestingly, age‐specific death and DALY rates of bladder cancer attributable to high FPG were found to have apparent increases among people younger than 39 years old, especially for males (Figure 3).

### 3.5 Association between SDI and bladder cancer burden attributable to high FPG

**FIGURE 4:**
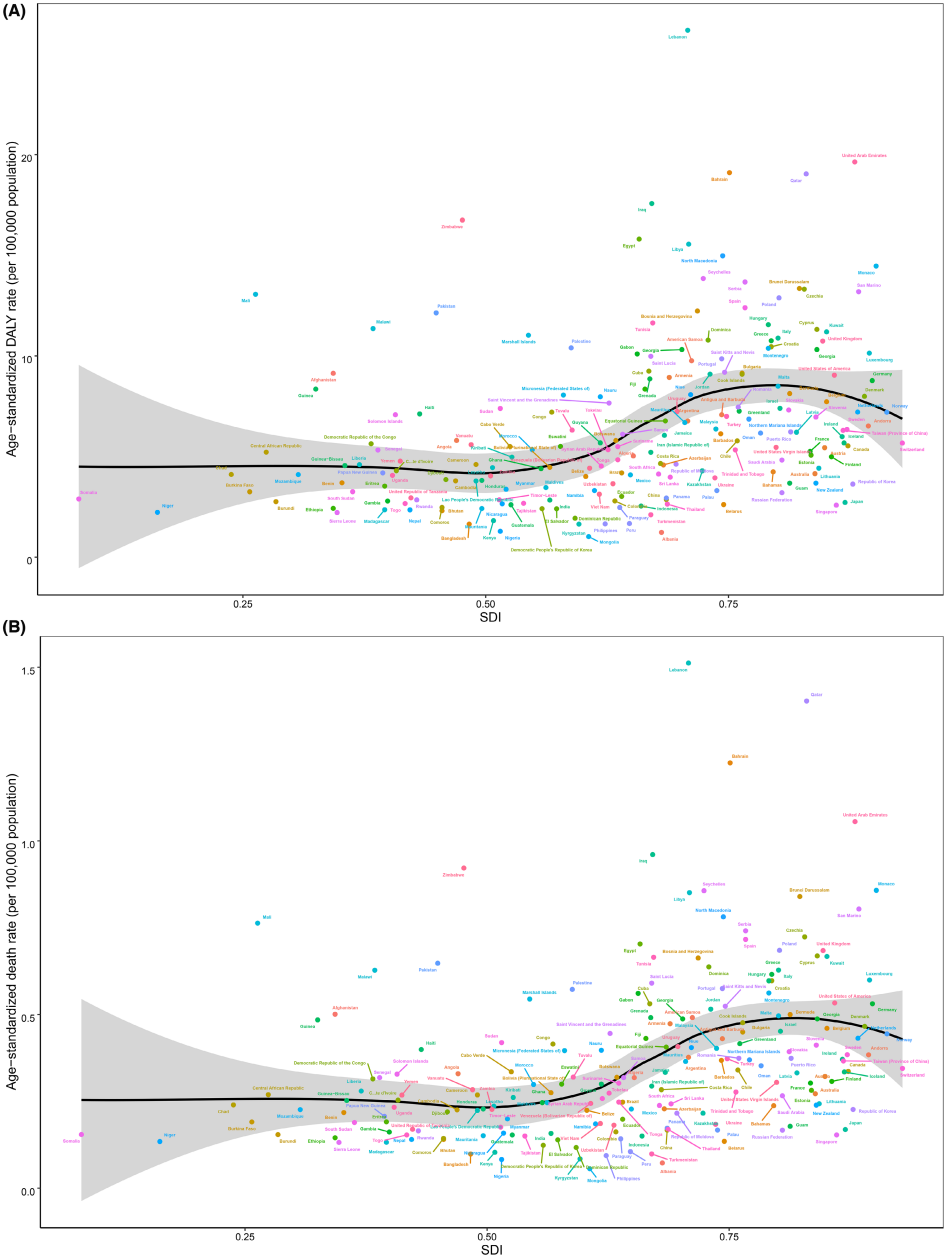
Associations between high FPG‐attributable age‐standardized rates (per 100,000 population) of bladder cancer and SDI among 204 countries and territories for both sexes combined. (A) age‐standardized DALY rates and SDI; (B) age‐standardized death rates and SDI. DALY, disability‐adjusted life‐year; FPG, fasting plasma glucose; SDI, sociodemographic index.

**FIGURE 5:**
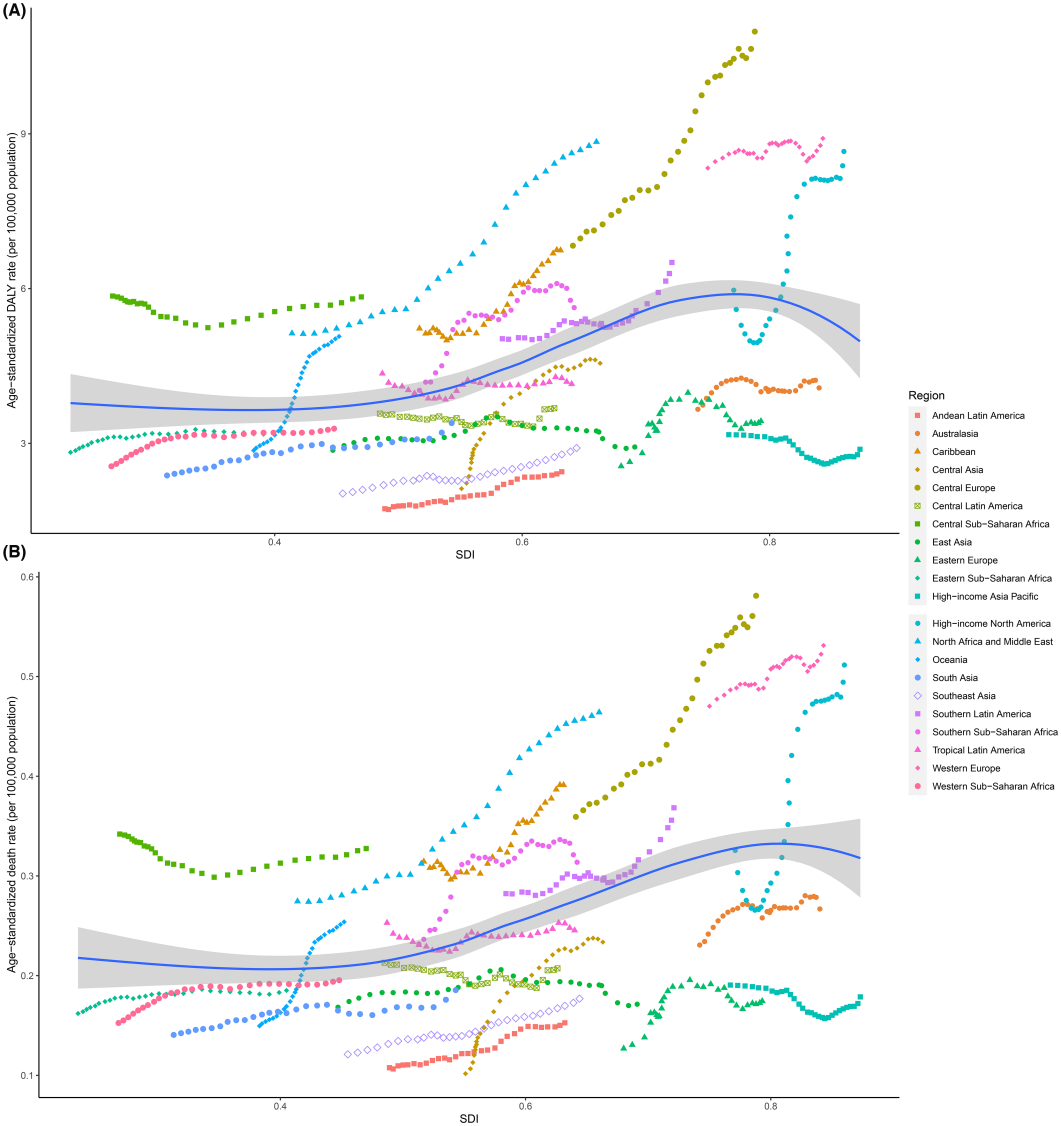
Associations between high FPG‐attributable age‐standardized rates (per 100,000 population) of bladder cancer and SDI among regions for both sexes combined. (A) age‐standardized DALY rates and SDI; (B) age‐standardized death rates and SDI. DALY, disability‐adjusted life‐year; FPG, fasting plasma glucose; SDI, sociodemographic index.

Bladder cancer burden attributable to high FPG varied greatly across regions and countries according to SDI. In 2019, high FPG‐associated age‐standardized death and DALY rates were more remarkable in countries with higher SDI, particularly Lebanon, and Qatar (Table 1, Figure 4, and Table S1). Age‐standardized death and DALY rates of bladder cancer attributable to high FPG were expected to rise with SDI when SDI was less than about 0.78 and then declined according to SDI (Figures 4 and 5). As Figure 5 illustrates, age‐standardized death and DALY rates of bladder cancer due to high FPG were higher than the expected level in several regions, including Central and Western Europe, North Africa and Middle East, and Central and Western Sub‐Saharan Africa. However, regional patterns showed great variations. Most of the regions presented increasing or remained unchanged age‐standardized death and DALY rates according to SDI, whereas some regions, such as Eastern Europe and High‐income North America, did not show monotonic relations with SDI (Figure 5).

Over the last 30 years, age‐standardized death, and DALY rates of bladder cancer attributable to high FPG have increased steadily in middle and lower SDI quintiles (Table 1 and Figure S3). Nonetheless, in higher SDI quintiles, the bladder cancer burden attributable to high FPG has been at a higher level despite fluctuations through the last three decades. More specifically, in countries with high SDI, high FPG‐attributable age‐standardized death, and DALY rates of bladder cancer fell in the first decade and then rose greatly until 2019. Among countries with high‐middle SDI, the rates rose rapidly from 1990 to 2005 and remained stable at their greatest level for the next 6 years then gradually drop until 2019.

## 4 DISCUSSION

In this study, we estimated the global level and trends of bladder cancer burden attributable to high FPG from 1990 to 2019. Across the world, the bladder cancer burden was reported to decrease during the past 30 years, which is in part due to advancements in cancer diagnosis and treatments, and the control of modifiable risks.^2^, ^13^ Among the risk factors, smoking is considered the most important contributor to the bladder cancer burden among all risk factors.^14^ Regarding occupational exposures, bladder cancer risk was found to be increased among tobacco workers and dye workers.^15^ Furthermore, several dietary factors, such as alcohol, vitamins, meat, fruits, and vegetables, were linked to bladder cancer risk, but the associations were conflicting and needed to be further validated.^16^, ^17^, ^18^ Due to complex agents in the workplace and confounding relations between dietary factors and lifestyles, it is difficult to assess the absolute effect of a single factor on bladder cancer risk. As for medical conditions, prior studies showed an increased risk of bladder cancer among diabetic patients, patients using anti‐diabetic drugs, and patients previously treated with pelvic radiation.^19^, ^20^ The level of FPG is associated with the diabetic condition, the prediabetic condition, and the effects of anti‐diabetic drugs. Therefore, smoking and high FPG were listed as risk factors for bladder cancer, and their effects on bladder cancer death and DALY were summarized in GBD studies. In line with our results, the smoking‐attributable bladder cancer burden was found to decrease since 1990.^2^ In contrast, high FPG resulted in 22.82 thousand deaths and 399.65 thousand DALYs of bladder cancer in 2019, with a great increase in bladder cancer burden worldwide during the past three decades. As our results presented, global high FPG‐related age‐standardized death and DALY rates of bladder cancer substantially rose by 39.18% and 41.48%, respectively, over the past 30 years. Meanwhile, the age‐standardized mean FPG was reported to increase from 1980 to 2008 and has become the fifth risk factor for all‐cause cancer in 2019,^6^, ^21^ implying the importance of controlling and monitoring FPG levels among patients with cancer.

Some studies have reported potential mechanisms for the association between bladder cancer burden and high FPG. First, high FPG can cause hyperglycemia that leads to the accumulation of glycation end‐products and chronic oxidative stress, which might produce reactive oxygen species to damage DNA.^22^, ^23^ Second, it might induce insulin resistance to facilitate tumor growth, resulting in worse cancer outcomes.^24^ Moreover, there were potential joint effects of high FPG and other risk factors, such as smoking and obesity. The high FPG interacting with obesity may alter the prognosis of patients with cancer.^25^ Smoking was previously reported to induce impaired fasting glucose.^26^ Meanwhile, smoking could result in a long‐term reduction of insulin and increase the fasting glucose level in men but decrease the level of insulin and fasting glucose in women.^27^ However, some studies also demonstrated there was no significant relation between smoking and the level of FPG.^28^ Thus, the relation between smoking and FPG levels should be explored further and validated in future studies. As mentioned above, a great amount of evidence also suggested that anti‐diabetes agents may play an important role in bladder cancer development. One study suggested that there was a significantly increased risk of bladder cancer among patients with diabetes using oral antidiabetic therapy, but no significance was observed between the patients with diabetes using diet‐controlled therapy.^29^ The anti‐diabetes agent, Metformin, has been demonstrated to act an anti‐cancer role in cancer development,^30^, ^31^ whereas rosiglitazone and pioglitazone were found to be associated with the risk of bladder cancer.^32^ All these findings cannot completely explain the association between bladder cancer development and high FPG or diabetes condition. Therefore, with the increasing hazard of high FPG worldwide, further investigation focused on how high FPG, diabetes conditions, and anti‐diabetic drugs act on bladder cancer development is necessary.

In our study, the increase in high FPG‐associated bladder cancer burden was more remarkable in males compared with their female counterparts. In line with our results, a male predominance was validated in bladder cancer burden, with consistently almost triple incident cases and deaths in males compared with females in 2019.^2^ At the same time, the FPG level was found to be higher in males than in females, and such elevated FPG level was linked to an increased risk of all‐cause mortality.^33^, ^34^ Among the elderly, bladder cancer deaths and DALYs due to high FPG were also higher, consistent with age distributions of high FPG‐associated all‐cause burden.^4^ Meanwhile, in our study, we also found obvious increases in age‐specific death and DALY rates of bladder cancer attributable to high FPG among young people aged 25–39 years, particularly in young males. Thus, FPG levels among older people with bladder cancer should be monitored more closely, and the FPG levels among younger people should also be paid better attention to.

High FPG‐attributable bladder cancer burden varied substantially among regions and countries. As our results showed, age‐standardized death and DALY rates of bladder cancer attributable to high FPG were more considerable in higher SDI quintiles. Similarly, a positive relation between SDI and all cancer burdens due to high FPG was also observed.^11^ Consistent with the previous study, high FPG‐attributable age‐standardized death and DALY rates of bladder cancer achieved their highest levels among countries in Central and Western Europe, and in North Africa and Middle East.^3^ Previous study also suggested that people with diabetes living in the more developed region showed a higher risk of bladder cancer.^35^ However, during the last 30 years, the largest increase in bladder cancer burden attributable to high FPG was observed in the lower‐middle SDI quintile. Several possible reasons might be helpful to interpret this result. First, although a healthy diet was more common among people with higher education in high‐income countries, the intake of higher caloric foods and a shift in work, from occupations highly requiring physical activity such as agriculture to sedentary jobs, contributed to a higher prevalence of obesity and increased the FPG level and the risk of cancer morbidity and mortality in these countries.^36^, ^37^, ^38^ Second, patients with cancer may be more likely to be diagnosed in higher‐income countries, with improved medical management and advanced cancer screening systems.^39^ Adversely, limited equipment and cancer registries, and a lack of community awareness of the elevated risk of cancers owing to diabetes, might result in a lower cancer burden attributable to high FPG in lower SDI countries.^40^ Moreover, genetic variations correlated with high FPG and diabetes among populations from different countries could also partially reflect the varied patterns of bladder cancer burden due to high FPG across the world,^41^, ^42^ which however was not analyzed in the present study. Except the potential reasons mentioned above, other factors that caused the difference between populations among countries should be explored further. Therefore, collaborations among countries are highly supported to improve the cancer care and management in countries with lower SDI. Meanwhile, the importance of healthy diet and physical activity should be strengthened, and effective interventions in these modifiable risks should be established in both high and low SDI countries, to reduce the heavy and increasing burden caused by high FPG.

Compared with previous studies,^4^, ^11^ this study provided the first and the most comprehensive analysis of the global bladder cancer burden attributable to high FPG from 1990 to 2019. We also provided detailed information on the bladder cancer burden attributable to high FPG among regions and countries for local governments to allocate resources and implement more effective measures. Despite great efforts, our study still exists several limitations. First, the high FPG‐associated bladder cancer burden might be underestimated in lower SDI countries where fewer people could access medical facilities and are covered by cancer registration systems.^43^ In GBD studies, the data from different resources were standardized, and all estimates were reported with their respective levels of uncertainty.^5^ Despite this, the findings need to be taken with care. Second, owing to a lack of corresponding data, a more specific analysis of bladder cancer subtypes due to high FPG and the effects of anti‐diabetic therapies on bladder cancer burden was not conducted in the present study. Third, inherited variations can greatly affect the FPG level and diabetes conditions, further contributing to the differences in population across countries. Therefore, owing to the lack of specific information regarding genetic variations, pathological features, and treatments of patients in current GBD studies, more research may gather and analyze the relevant data in the future.

## 5 CONCLUSION

To conclude, our study documents a higher level of bladder cancer burden attributable to high FPG among the elderly, males, and people in countries with higher SDI. Meanwhile, the high FPG‐associated bladder cancer burden presented a markedly upward trend in all countries. Therefore, our study provides detailed evidence regarding the global bladder cancer burden due to high levels of FPG and can assist in implementing effective measures, allocating resources, and facilitating collaborations between countries. Practically for the time being, increased attention should be made to the monitoring and management of FPG levels among bladder cancer patients to lessen the growing burden. In future, we strongly urge the development of more policies to promote healthy lifestyles and the pursuit of studies on potential mechanisms and effects of high FPG on bladder cancer risk.

## AUTHOR CONTRIBUTIONS

**Ying Wu:** Methodology (lead); software (lead); visualization (lead); writing – original draft (lead). **Yu‐Jiao Deng:** Investigation (lead); methodology (supporting). **Zhi‐Jun Dai:** Writing – review and editing (supporting). **Yubo Ma:** Investigation (equal); methodology (supporting). **Lijuan Lyu:** Investigation (supporting); visualization (supporting). **Chen Lei:** Writing – review and editing (supporting). **Yi Zheng:** Investigation (supporting); visualization (supporting). **Yizhen Li:** Investigation (supporting); visualization (supporting). **Ziming Wang:** Conceptualization (lead); formal analysis (lead). **Jie Gao:** Conceptualization (lead); data curation (lead); formal analysis (lead).

## CONFLICT OF INTEREST STATEMENT

The authors declare no potential conflicts of interest.

## ETHICS STATEMENT

The ethical consent of GBD studies was waivered and approved by the Institutional Reviewing Board of University of Washington. All the data and the other detailed information are accessible via the GBD official website and its query tool.

## Supporting information


Figure S1.
Figure S2.
Figure S3.
Table S1.
Table S2.

## Data Availability

Availability of data and materialsAll data are accessible through the GBD query tool (http://www.healthdata.org/gbd/2019).
