# Supplementary material for: Estimates of bladder cancer burden attributable to high fasting plasma glucose: Findings of the Global Burden of Disease Study 2019
Source: Cancer Med. 2023 Jun 23;12(15):16469–81. doi: 10.1002/cam4.6219 (PMC10469723; doi:10.1002/cam4.6219)
Supplement: Supplementary file 1 — Figure S1. Figure S2. Figure S3. Table S1. Table S2. [file CAM4-12-16469-s001.docx]

**Table of content**

[Figure S1 2](#_Toc120010621)

[Figure S2 3](#_Toc120010622)

[Figure S3 4](#_Toc120010623)

[Table S1 5](#_Toc120010624)

[Table S2 14](#_Toc120010625)


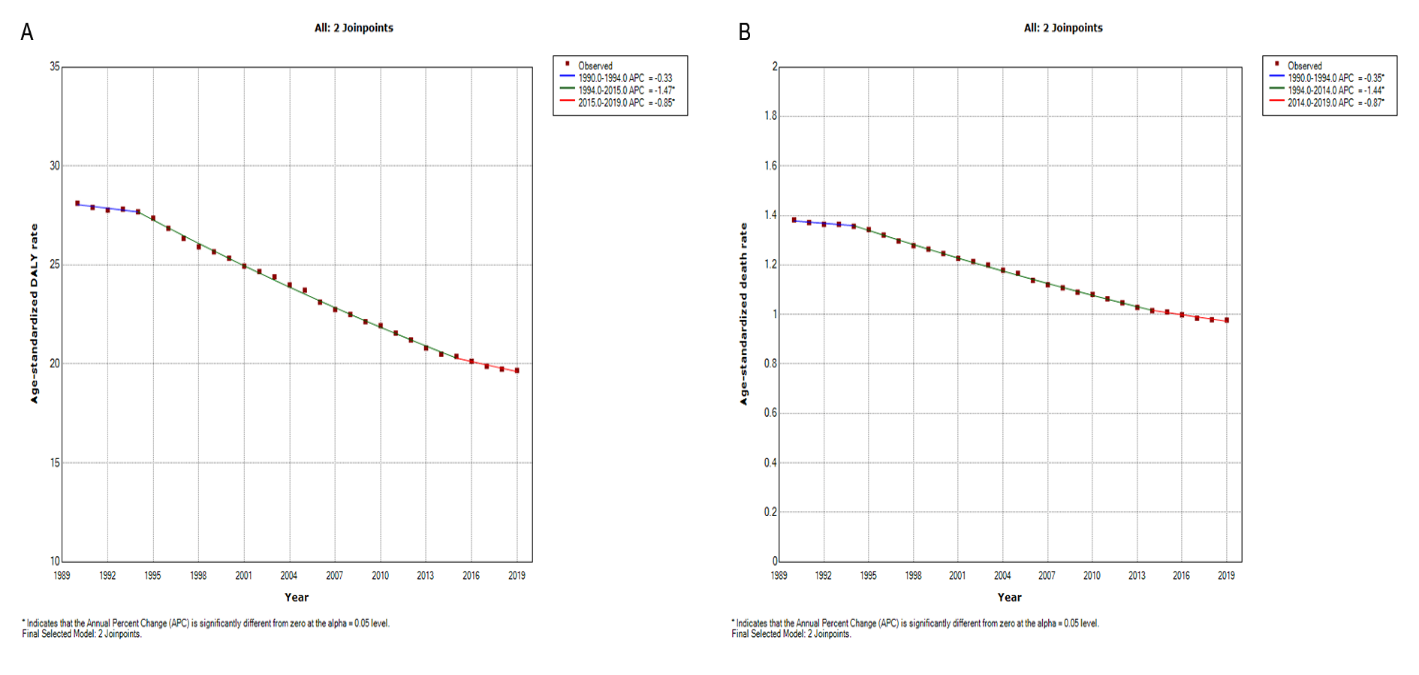


Figure S1 **Global trend of bladder cancer burden attributable to smoking from 1990 to 2019.** (A) age-standardized DALY rate; (B) age-standardized death rate.

Abbreviations: DALY disability-adjusted life years.


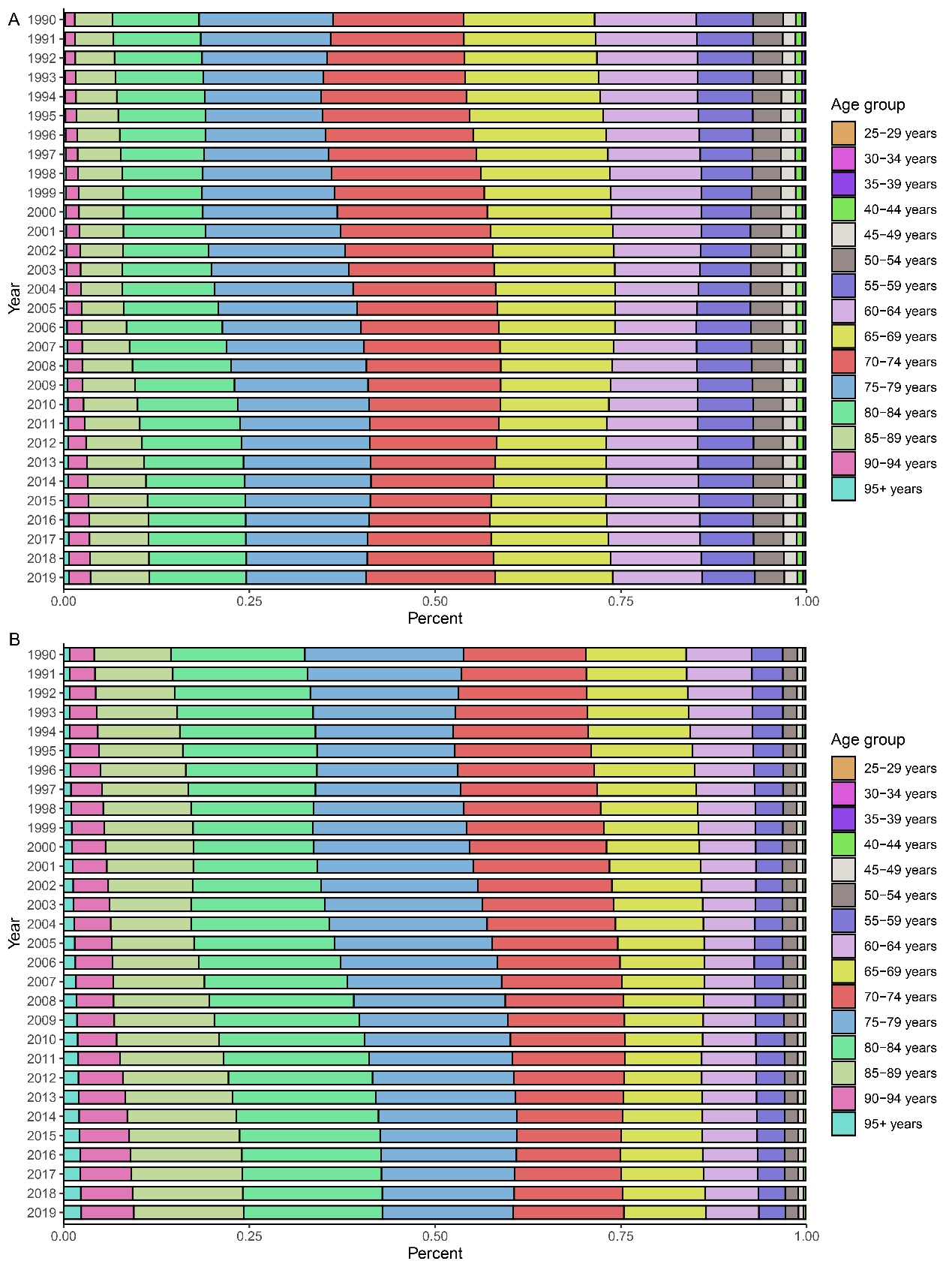


Figure S2 **Burdens of bladder cancer attributable to high fasting plasma glucose in different age groups for both sexes combined from 1990 to 2019.** (A) DALY; (B) death.

Abbreviations: DALYs disability-adjusted life years.


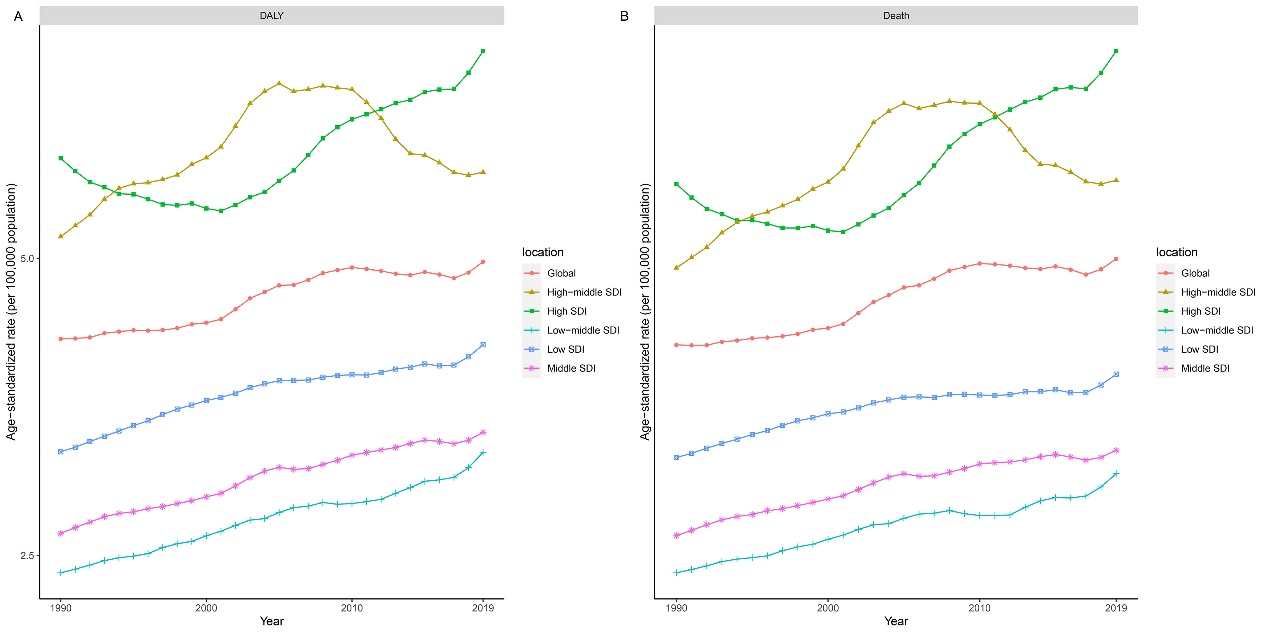


Figure S3 **Trends for age-standardized rates (per 100,000 population) of bladder cancer attributable to high FPG among five SDI quintiles for both sexes combined from 1990 to 2019.** (A) age-standardized DALY rate; (B) age-standardized death rate.

Abbreviations: SDI sociodemographic index; DALY disability-adjusted life-year; FPG fasting plasma glucose.

Table S1 **National burden of bladder cancer attributable to high FPG in 2019**

| location | ASDR in 2019 | | | Age-standardized DALY rate in 2019 | | |
| --- | --- | --- | --- | --- | --- | --- |
|  | Both | Female | Male | Both | Female | Male |
| Afghanistan | 0.5(0.1 to 1.16) | 0.31(0.05 to 0.82) | 0.71(0.12 to 1.66) | 9.14(1.84 to 21.52) | 5.94(0.97 to 16.07) | 12.76(2.19 to 30.18) |
| Albania | 0.07(0.01 to 0.18) | 0.05(0.01 to 0.12) | 0.1(0.02 to 0.26) | 1.23(0.25 to 3.01) | 0.77(0.13 to 1.96) | 1.75(0.28 to 4.4) |
| Algeria | 0.32(0.07 to 0.72) | 0.14(0.02 to 0.31) | 0.47(0.09 to 1.08) | 5.03(1.08 to 11.42) | 2.11(0.37 to 4.76) | 7.73(1.41 to 17.92) |
| American Samoa | 0.49(0.12 to 0.96) | 0.4(0.08 to 0.87) | 0.59(0.12 to 1.22) | 9.76(2.41 to 19.41) | 7.9(1.58 to 17.16) | 11.79(2.37 to 24.39) |
| Andorra | 0.38(0.08 to 0.89) | 0.08(0.01 to 0.2) | 0.74(0.13 to 1.71) | 6.52(1.25 to 15.23) | 1.27(0.21 to 3.19) | 12.01(2.19 to 28.57) |
| Angola | 0.33(0.06 to 0.96) | 0.12(0.02 to 0.37) | 0.64(0.1 to 1.94) | 5.8(1.06 to 16.56) | 2.07(0.32 to 6.2) | 10.8(1.64 to 32.13) |
| Antigua and Barbuda | 0.43(0.1 to 0.9) | 0.35(0.07 to 0.79) | 0.53(0.09 to 1.15) | 7.08(1.66 to 15.06) | 5.86(1.08 to 13.2) | 8.55(1.54 to 18.77) |
| Argentina | 0.38(0.07 to 0.82) | 0.14(0.03 to 0.33) | 0.72(0.12 to 1.6) | 6.77(1.32 to 14.74) | 2.42(0.42 to 5.58) | 12.56(2.1 to 28.03) |
| Armenia | 0.47(0.09 to 1.07) | 0.11(0.02 to 0.26) | 1.02(0.17 to 2.34) | 8.93(1.65 to 20.4) | 1.98(0.34 to 4.69) | 18.74(3.12 to 43.12) |
| Australia | 0.27(0.05 to 0.59) | 0.12(0.02 to 0.29) | 0.47(0.07 to 1.07) | 4.15(0.78 to 9.1) | 1.79(0.31 to 4.28) | 6.97(1.14 to 15.91) |
| Austria | 0.32(0.06 to 0.73) | 0.14(0.02 to 0.33) | 0.59(0.1 to 1.39) | 5.44(1.09 to 12.3) | 2.26(0.39 to 5.25) | 9.62(1.6 to 22.28) |
| Azerbaijan | 0.23(0.05 to 0.52) | 0.09(0.01 to 0.21) | 0.42(0.07 to 0.99) | 4.58(0.91 to 10.44) | 1.59(0.28 to 3.82) | 8.43(1.34 to 20.1) |
| Bahamas | 0.24(0.05 to 0.52) | 0.14(0.02 to 0.32) | 0.38(0.07 to 0.84) | 4.24(0.91 to 9.32) | 2.42(0.43 to 5.73) | 6.58(1.13 to 14.85) |
| Bahrain | 1.22(0.31 to 2.45) | 0.54(0.11 to 1.09) | 1.88(0.39 to 3.89) | 19.11(4.7 to 38.28) | 7.72(1.64 to 15.79) | 28.83(6.11 to 60.25) |
| Bangladesh | 0.1(0.02 to 0.23) | 0.05(0.01 to 0.14) | 0.14(0.02 to 0.35) | 1.64(0.32 to 4) | 0.89(0.15 to 2.22) | 2.32(0.37 to 5.92) |
| Barbados | 0.37(0.08 to 0.78) | 0.24(0.04 to 0.56) | 0.54(0.1 to 1.19) | 6.13(1.41 to 13.15) | 4.01(0.74 to 9.21) | 8.8(1.6 to 19.76) |
| Belarus | 0.13(0.03 to 0.33) | 0.04(0.01 to 0.1) | 0.34(0.05 to 0.83) | 2.61(0.47 to 6.42) | 0.66(0.1 to 1.74) | 6.15(0.97 to 15.36) |
| Belgium | 0.46(0.09 to 1.01) | 0.19(0.03 to 0.44) | 0.85(0.14 to 1.96) | 7.72(1.47 to 17.08) | 2.94(0.55 to 6.97) | 13.79(2.25 to 31.38) |
| Belize | 0.22(0.05 to 0.5) | 0.19(0.03 to 0.44) | 0.26(0.04 to 0.59) | 4.01(0.88 to 9) | 3.42(0.61 to 7.87) | 4.58(0.75 to 10.54) |
| Benin | 0.22(0.04 to 0.5) | 0.11(0.02 to 0.28) | 0.36(0.06 to 0.86) | 3.7(0.77 to 8.39) | 1.92(0.31 to 4.87) | 5.92(0.99 to 14.12) |
| Bermuda | 0.5(0.1 to 1.11) | 0.17(0.03 to 0.42) | 0.98(0.17 to 2.2) | 8.13(1.58 to 18.21) | 2.55(0.45 to 6.27) | 15.56(2.67 to 35) |
| Bhutan | 0.14(0.03 to 0.33) | 0.08(0.01 to 0.2) | 0.2(0.03 to 0.5) | 2.31(0.47 to 5.53) | 1.31(0.23 to 3.31) | 3.27(0.51 to 8.35) |
| Bolivia (Plurinational State of) | 0.27(0.06 to 0.61) | 0.18(0.03 to 0.45) | 0.38(0.06 to 0.9) | 4.46(0.96 to 10.05) | 3.06(0.54 to 7.6) | 6.08(1.02 to 14.49) |
| Bosnia and Herzegovina | 0.66(0.14 to 1.48) | 0.25(0.05 to 0.58) | 1.25(0.23 to 2.8) | 12.23(2.59 to 27.21) | 4.38(0.86 to 10.38) | 22.57(4.14 to 51) |
| Botswana | 0.32(0.07 to 0.7) | 0.19(0.03 to 0.45) | 0.56(0.09 to 1.26) | 5.8(1.26 to 12.93) | 3.4(0.55 to 8.45) | 9.61(1.65 to 21.97) |
| Brazil | 0.25(0.05 to 0.54) | 0.13(0.02 to 0.3) | 0.41(0.07 to 0.93) | 4.19(0.89 to 9.16) | 2.25(0.41 to 5.14) | 6.73(1.07 to 15.35) |
| Brunei Darussalam | 0.84(0.2 to 1.68) | 0.45(0.09 to 0.97) | 1.76(0.36 to 3.63) | 13.35(3.24 to 26.92) | 7.12(1.43 to 15.3) | 24.79(5.25 to 51.15) |
| Bulgaria | 0.45(0.09 to 1.03) | 0.15(0.03 to 0.35) | 0.89(0.15 to 2.06) | 9.14(1.7 to 21) | 2.74(0.47 to 6.72) | 17.7(3.02 to 40.48) |
| Burkina Faso | 0.19(0.04 to 0.46) | 0.1(0.02 to 0.27) | 0.31(0.05 to 0.8) | 3.25(0.65 to 7.79) | 1.77(0.24 to 4.69) | 5.13(0.78 to 13.14) |
| Burundi | 0.15(0.03 to 0.37) | 0.1(0.01 to 0.28) | 0.21(0.03 to 0.55) | 2.76(0.56 to 6.85) | 1.78(0.26 to 5.13) | 3.72(0.59 to 9.72) |
| Cabo Verde | 0.34(0.07 to 0.73) | 0.15(0.03 to 0.33) | 0.64(0.12 to 1.51) | 5.5(1.14 to 12.27) | 2.38(0.42 to 5.43) | 10.12(1.75 to 23.75) |
| Cambodia | 0.23(0.05 to 0.51) | 0.11(0.02 to 0.28) | 0.41(0.08 to 0.99) | 3.79(0.81 to 8.64) | 1.89(0.33 to 4.73) | 6.78(1.23 to 16.42) |
| Cameroon | 0.27(0.05 to 0.63) | 0.13(0.02 to 0.32) | 0.45(0.07 to 1.09) | 4.61(0.92 to 10.91) | 2.18(0.3 to 5.61) | 7.43(1.16 to 18.33) |
| Canada | 0.34(0.07 to 0.75) | 0.13(0.02 to 0.31) | 0.6(0.1 to 1.38) | 5.53(1.04 to 12.37) | 2.2(0.38 to 5.18) | 9.53(1.55 to 21.86) |
| Central African Republic | 0.27(0.05 to 0.66) | 0.12(0.02 to 0.34) | 0.53(0.09 to 1.34) | 5.22(0.98 to 12.45) | 2.2(0.34 to 6.58) | 9.71(1.62 to 24.25) |
| Chad | 0.24(0.05 to 0.57) | 0.12(0.02 to 0.29) | 0.35(0.05 to 0.88) | 4.11(0.79 to 9.83) | 2.05(0.35 to 5.25) | 5.86(0.93 to 15.01) |
| Chile | 0.34(0.07 to 0.73) | 0.21(0.04 to 0.47) | 0.52(0.09 to 1.15) | 5.78(1.28 to 12.29) | 3.46(0.62 to 7.75) | 8.73(1.51 to 19.54) |
| China | 0.17(0.03 to 0.37) | 0.06(0.01 to 0.15) | 0.33(0.05 to 0.76) | 2.87(0.55 to 6.37) | 1.04(0.18 to 2.47) | 5.18(0.84 to 12.12) |
| Colombia | 0.16(0.04 to 0.37) | 0.1(0.02 to 0.24) | 0.24(0.04 to 0.55) | 2.79(0.62 to 6.41) | 1.71(0.31 to 4.15) | 4.13(0.72 to 9.58) |
| Comoros | 0.14(0.03 to 0.35) | 0.09(0.01 to 0.24) | 0.21(0.03 to 0.56) | 2.47(0.45 to 6.09) | 1.65(0.22 to 4.29) | 3.53(0.48 to 9.63) |
| Congo | 0.42(0.08 to 1.07) | 0.18(0.03 to 0.49) | 0.73(0.12 to 1.85) | 7.21(1.44 to 18.21) | 3.15(0.47 to 8.77) | 12.11(2.04 to 30.63) |
| Cook Islands | 0.48(0.12 to 1.01) | 0.45(0.08 to 1.02) | 0.5(0.1 to 1.06) | 9.09(2.18 to 19.36) | 8.17(1.52 to 18.87) | 9.97(1.88 to 21.54) |
| Costa Rica | 0.28(0.06 to 0.64) | 0.12(0.02 to 0.28) | 0.49(0.09 to 1.1) | 4.65(0.96 to 10.47) | 1.92(0.35 to 4.61) | 7.93(1.43 to 17.85) |
| Croatia | 0.6(0.12 to 1.32) | 0.23(0.04 to 0.54) | 1.21(0.21 to 2.72) | 10.45(2.07 to 23.76) | 3.69(0.67 to 8.86) | 20.16(3.57 to 46.62) |
| Cuba | 0.53(0.11 to 1.15) | 0.22(0.04 to 0.5) | 0.91(0.16 to 2.03) | 9.24(1.9 to 20.03) | 3.74(0.64 to 8.81) | 15.61(2.81 to 35.01) |
| Cyprus | 0.67(0.15 to 1.46) | 0.21(0.04 to 0.48) | 1.24(0.22 to 2.75) | 11.32(2.49 to 24.7) | 3.12(0.57 to 7.1) | 20.75(3.67 to 46.52) |
| Czechia | 0.72(0.15 to 1.55) | 0.29(0.06 to 0.67) | 1.38(0.25 to 3.01) | 13.32(2.8 to 28.73) | 5.17(0.99 to 11.94) | 24.29(4.46 to 53.68) |
| Côte d'Ivoire | 0.25(0.05 to 0.58) | 0.12(0.02 to 0.3) | 0.4(0.07 to 0.95) | 4.3(0.86 to 10.14) | 2.07(0.34 to 4.95) | 6.54(1.15 to 15.85) |
| Democratic People's Republic of Korea | 0.12(0.02 to 0.29) | 0.07(0.01 to 0.16) | 0.25(0.04 to 0.61) | 2.41(0.5 to 5.56) | 1.24(0.19 to 3.07) | 4.45(0.73 to 10.77) |
| Democratic Republic of the Congo | 0.31(0.05 to 0.81) | 0.12(0.02 to 0.33) | 0.64(0.1 to 1.74) | 5.63(0.97 to 14.51) | 2.13(0.33 to 5.91) | 10.78(1.63 to 28.92) |
| Denmark | 0.47(0.09 to 1.02) | 0.24(0.04 to 0.56) | 0.76(0.12 to 1.75) | 7.99(1.57 to 17.77) | 4.02(0.68 to 9.38) | 12.72(2.03 to 29.14) |
| Djibouti | 0.22(0.04 to 0.53) | 0.13(0.02 to 0.32) | 0.31(0.05 to 0.8) | 3.85(0.77 to 9.74) | 2.25(0.36 to 5.72) | 5.34(0.8 to 14.1) |
| Dominica | 0.64(0.15 to 1.36) | 0.46(0.08 to 1.04) | 0.86(0.16 to 1.86) | 10.78(2.54 to 23.41) | 7.52(1.37 to 17.37) | 14.36(2.61 to 31.77) |
| Dominican Republic | 0.12(0.02 to 0.28) | 0.08(0.01 to 0.21) | 0.16(0.03 to 0.38) | 1.93(0.4 to 4.56) | 1.33(0.22 to 3.42) | 2.61(0.44 to 6.35) |
| Ecuador | 0.2(0.04 to 0.44) | 0.13(0.02 to 0.31) | 0.28(0.05 to 0.65) | 3.02(0.66 to 6.85) | 1.99(0.35 to 4.81) | 4.2(0.69 to 9.83) |
| Egypt | 0.7(0.13 to 1.73) | 0.3(0.05 to 0.76) | 1.02(0.16 to 2.56) | 15.8(2.8 to 39) | 6.35(0.96 to 16.07) | 23.72(3.59 to 60.05) |
| El Salvador | 0.14(0.03 to 0.32) | 0.09(0.02 to 0.23) | 0.2(0.03 to 0.46) | 2.4(0.53 to 5.49) | 1.6(0.29 to 3.95) | 3.5(0.6 to 8.06) |
| Equatorial Guinea | 0.41(0.08 to 0.98) | 0.16(0.03 to 0.41) | 0.8(0.14 to 2.02) | 6.77(1.37 to 16.31) | 2.64(0.41 to 7.14) | 12.95(2.25 to 33.02) |
| Eritrea | 0.19(0.04 to 0.46) | 0.15(0.02 to 0.39) | 0.28(0.04 to 0.71) | 3.53(0.72 to 8.67) | 2.69(0.4 to 7.1) | 4.98(0.79 to 12.82) |
| Estonia | 0.28(0.06 to 0.66) | 0.1(0.02 to 0.25) | 0.65(0.11 to 1.58) | 5.07(1 to 11.98) | 1.66(0.27 to 4.09) | 11.07(1.83 to 26.86) |
| Eswatini | 0.3(0.06 to 0.67) | 0.17(0.03 to 0.43) | 0.57(0.1 to 1.33) | 5.5(1.18 to 12.6) | 3.05(0.5 to 7.85) | 10.07(1.66 to 23.51) |
| Ethiopia | 0.15(0.03 to 0.36) | 0.08(0.01 to 0.21) | 0.21(0.03 to 0.55) | 2.43(0.45 to 6) | 1.33(0.21 to 3.63) | 3.45(0.5 to 9.09) |
| Fiji | 0.43(0.11 to 0.88) | 0.26(0.05 to 0.6) | 0.68(0.14 to 1.4) | 8.35(2.08 to 17.49) | 5.24(1.05 to 12) | 12.36(2.5 to 25.85) |
| Finland | 0.31(0.06 to 0.66) | 0.14(0.03 to 0.32) | 0.56(0.09 to 1.24) | 4.99(1.01 to 10.93) | 2.17(0.41 to 4.96) | 8.68(1.44 to 19.61) |
| France | 0.3(0.06 to 0.7) | 0.1(0.02 to 0.25) | 0.59(0.09 to 1.39) | 5.26(0.94 to 12.07) | 1.59(0.26 to 3.86) | 9.95(1.57 to 23.26) |
| Gabon | 0.56(0.11 to 1.63) | 0.19(0.03 to 0.67) | 1.14(0.17 to 3.51) | 10.1(1.83 to 29.07) | 3.19(0.47 to 11.04) | 19.33(2.99 to 59.53) |
| Gambia | 0.16(0.03 to 0.38) | 0.1(0.02 to 0.24) | 0.24(0.04 to 0.6) | 2.79(0.54 to 6.52) | 1.72(0.28 to 4.3) | 4.03(0.65 to 10.14) |
| Georgia | 0.49(0.09 to 1.09) | 0.13(0.02 to 0.29) | 1.06(0.19 to 2.43) | 10.31(1.94 to 23.08) | 2.37(0.42 to 5.48) | 21.78(3.89 to 49.86) |
| Germany | 0.53(0.11 to 1.13) | 0.25(0.05 to 0.55) | 0.94(0.16 to 2.06) | 8.77(1.86 to 18.89) | 3.88(0.72 to 8.75) | 15.01(2.53 to 32.82) |
| Ghana | 0.25(0.05 to 0.57) | 0.13(0.02 to 0.33) | 0.42(0.07 to 1.01) | 4.4(0.86 to 10.48) | 2.22(0.35 to 5.59) | 7.37(1.11 to 18.1) |
| Greece | 0.62(0.12 to 1.36) | 0.16(0.03 to 0.38) | 1.18(0.19 to 2.64) | 10.75(2.02 to 24.02) | 2.52(0.44 to 5.93) | 20.57(3.34 to 46.4) |
| Greenland | 0.42(0.08 to 0.95) | 0.13(0.02 to 0.32) | 0.71(0.12 to 1.64) | 7.26(1.35 to 16.63) | 2.03(0.34 to 4.98) | 12.11(1.96 to 27.91) |
| Grenada | 0.49(0.12 to 1.02) | 0.3(0.06 to 0.66) | 0.78(0.15 to 1.69) | 8.86(2.05 to 18.6) | 5.16(0.96 to 11.38) | 13.52(2.56 to 29.43) |
| Guam | 0.18(0.04 to 0.39) | 0.09(0.02 to 0.21) | 0.28(0.05 to 0.65) | 3.68(0.76 to 8.21) | 1.74(0.3 to 4.19) | 5.76(0.99 to 13.25) |
| Guatemala | 0.15(0.04 to 0.34) | 0.11(0.02 to 0.26) | 0.21(0.04 to 0.46) | 2.6(0.6 to 5.77) | 1.96(0.38 to 4.61) | 3.42(0.59 to 7.73) |
| Guinea | 0.48(0.09 to 1.17) | 0.18(0.03 to 0.45) | 0.8(0.13 to 1.99) | 8.35(1.55 to 20.36) | 3.25(0.5 to 8.09) | 13.35(2.17 to 33.86) |
| Guinea-Bissau | 0.25(0.05 to 0.57) | 0.14(0.02 to 0.35) | 0.42(0.07 to 0.98) | 4.55(0.91 to 10.39) | 2.5(0.4 to 6.4) | 7.29(1.2 to 17.3) |
| Guyana | 0.3(0.07 to 0.64) | 0.18(0.03 to 0.4) | 0.46(0.08 to 1.01) | 5.68(1.3 to 12.42) | 3.28(0.59 to 7.62) | 8.53(1.55 to 19.1) |
| Haiti | 0.4(0.08 to 0.98) | 0.31(0.05 to 0.85) | 0.5(0.08 to 1.21) | 7.12(1.46 to 17.81) | 5.82(0.93 to 16.31) | 8.58(1.41 to 21.03) |
| Honduras | 0.23(0.05 to 0.55) | 0.14(0.02 to 0.32) | 0.33(0.06 to 0.85) | 3.81(0.8 to 9.29) | 2.3(0.38 to 5.59) | 5.52(0.96 to 13.92) |
| Hungary | 0.6(0.12 to 1.32) | 0.24(0.04 to 0.57) | 1.21(0.21 to 2.67) | 11.55(2.26 to 25.9) | 4.62(0.79 to 11.06) | 22.09(3.81 to 49.41) |
| Iceland | 0.33(0.06 to 0.75) | 0.12(0.02 to 0.29) | 0.6(0.1 to 1.39) | 5.67(1.09 to 12.81) | 1.87(0.32 to 4.53) | 9.98(1.67 to 22.93) |
| India | 0.16(0.03 to 0.34) | 0.09(0.01 to 0.2) | 0.24(0.04 to 0.54) | 2.84(0.6 to 6.18) | 1.5(0.26 to 3.5) | 4.3(0.72 to 9.71) |
| Indonesia | 0.15(0.03 to 0.36) | 0.08(0.01 to 0.19) | 0.25(0.04 to 0.62) | 2.54(0.48 to 6.12) | 1.24(0.19 to 3.19) | 4.13(0.64 to 10.66) |
| Iran (Islamic Republic of) | 0.29(0.06 to 0.63) | 0.15(0.03 to 0.32) | 0.44(0.08 to 0.99) | 5.21(1.08 to 11.27) | 2.35(0.43 to 5.15) | 8.11(1.42 to 18.22) |
| Iraq | 0.96(0.2 to 2.04) | 0.42(0.08 to 0.98) | 1.59(0.29 to 3.48) | 17.57(3.57 to 37.69) | 7.83(1.46 to 18.24) | 28.34(5.21 to 62.28) |
| Ireland | 0.37(0.07 to 0.82) | 0.18(0.03 to 0.42) | 0.62(0.1 to 1.41) | 5.99(1.15 to 13.54) | 2.87(0.49 to 6.77) | 9.68(1.58 to 22.44) |
| Israel | 0.45(0.09 to 0.99) | 0.18(0.03 to 0.42) | 0.8(0.13 to 1.83) | 7.4(1.5 to 16.21) | 2.83(0.54 to 6.5) | 13(2.13 to 29.79) |
| Italy | 0.63(0.13 to 1.37) | 0.2(0.04 to 0.44) | 1.25(0.21 to 2.77) | 10.89(2.2 to 23.72) | 3.17(0.59 to 7.14) | 20.77(3.53 to 46.38) |
| Jamaica | 0.33(0.07 to 0.74) | 0.23(0.04 to 0.53) | 0.45(0.08 to 1.04) | 6.05(1.32 to 13.34) | 4.09(0.77 to 9.6) | 8.19(1.44 to 18.79) |
| Japan | 0.17(0.03 to 0.39) | 0.07(0.01 to 0.16) | 0.32(0.05 to 0.76) | 2.72(0.51 to 6.25) | 0.93(0.15 to 2.23) | 5.01(0.81 to 11.88) |
| Jordan | 0.52(0.1 to 1.12) | 0.14(0.03 to 0.33) | 0.86(0.16 to 1.95) | 9.09(1.81 to 20.1) | 2.32(0.42 to 5.48) | 15.35(2.76 to 35.25) |
| Kazakhstan | 0.22(0.04 to 0.48) | 0.08(0.01 to 0.18) | 0.47(0.07 to 1.1) | 4.29(0.84 to 9.73) | 1.46(0.26 to 3.4) | 8.95(1.41 to 21.15) |
| Kenya | 0.1(0.02 to 0.24) | 0.06(0.01 to 0.14) | 0.17(0.03 to 0.41) | 1.81(0.36 to 4.3) | 0.98(0.16 to 2.52) | 2.87(0.43 to 7.09) |
| Kiribati | 0.26(0.06 to 0.55) | 0.2(0.04 to 0.47) | 0.37(0.07 to 0.79) | 4.97(1.19 to 10.46) | 4.08(0.68 to 9.67) | 6.5(1.22 to 14.26) |
| Kuwait | 0.67(0.14 to 1.41) | 0.29(0.05 to 0.67) | 0.91(0.17 to 1.99) | 11.19(2.39 to 23.84) | 4.69(0.82 to 10.92) | 15.39(2.79 to 33.56) |
| Kyrgyzstan | 0.08(0.02 to 0.19) | 0.04(0.01 to 0.09) | 0.15(0.02 to 0.36) | 1.64(0.3 to 3.85) | 0.64(0.1 to 1.53) | 3.03(0.48 to 7.27) |
| Lao People's Democratic Republic | 0.22(0.05 to 0.49) | 0.12(0.02 to 0.3) | 0.35(0.06 to 0.81) | 3.79(0.78 to 8.51) | 2.04(0.34 to 5.13) | 5.8(1.01 to 13.71) |
| Latvia | 0.33(0.06 to 0.76) | 0.12(0.02 to 0.3) | 0.78(0.13 to 1.9) | 6.21(1.15 to 14.48) | 1.92(0.32 to 4.91) | 13.77(2.21 to 33.41) |
| Lebanon | 1.51(0.31 to 3.36) | 0.61(0.11 to 1.47) | 2.64(0.46 to 6) | 26.18(5.31 to 57.8) | 10.17(1.77 to 24.5) | 45.86(7.88 to 104.73) |
| Lesotho | 0.24(0.05 to 0.55) | 0.15(0.02 to 0.39) | 0.4(0.07 to 0.97) | 4.45(0.92 to 10.43) | 2.86(0.43 to 7.21) | 7.26(1.18 to 17.61) |
| Liberia | 0.28(0.06 to 0.68) | 0.14(0.02 to 0.36) | 0.42(0.07 to 1.03) | 4.6(0.92 to 11.17) | 2.48(0.4 to 6.27) | 6.7(1.14 to 16.61) |
| Libya | 0.85(0.17 to 1.9) | 0.25(0.05 to 0.58) | 1.44(0.25 to 3.29) | 15.55(3.06 to 34.93) | 4.77(0.84 to 11.07) | 26.02(4.58 to 60.1) |
| Lithuania | 0.24(0.04 to 0.57) | 0.07(0.01 to 0.18) | 0.58(0.09 to 1.42) | 4.42(0.83 to 10.66) | 1.22(0.19 to 2.98) | 10.02(1.56 to 24.94) |
| Luxembourg | 0.6(0.12 to 1.31) | 0.24(0.05 to 0.54) | 1.11(0.19 to 2.48) | 10.14(2.1 to 22.62) | 3.77(0.74 to 8.77) | 17.94(3.06 to 40.18) |
| Madagascar | 0.13(0.03 to 0.32) | 0.09(0.01 to 0.24) | 0.18(0.03 to 0.45) | 2.36(0.47 to 5.8) | 1.68(0.25 to 4.33) | 3.13(0.49 to 8.03) |
| Malawi | 0.63(0.12 to 1.53) | 0.4(0.04 to 1.02) | 0.98(0.12 to 2.59) | 11.36(2.13 to 28.2) | 7.08(0.77 to 18.07) | 17.43(2.28 to 46.63) |
| Malaysia | 0.4(0.08 to 0.89) | 0.19(0.03 to 0.44) | 0.62(0.11 to 1.41) | 6.37(1.3 to 14.24) | 2.99(0.54 to 7.07) | 9.86(1.66 to 22.47) |
| Maldives | 0.25(0.05 to 0.55) | 0.1(0.02 to 0.23) | 0.38(0.07 to 0.85) | 3.46(0.7 to 7.75) | 1.37(0.24 to 3.19) | 5.32(0.95 to 12.19) |
| Mali | 0.76(0.12 to 1.87) | 0.42(0.04 to 1.06) | 1.08(0.12 to 2.8) | 13.06(2.16 to 32.11) | 7.58(0.64 to 19.44) | 18.14(2.05 to 46.74) |
| Malta | 0.5(0.1 to 1.08) | 0.23(0.04 to 0.53) | 0.86(0.15 to 1.93) | 8.49(1.68 to 18.59) | 3.75(0.71 to 8.72) | 14.3(2.42 to 31.98) |
| Marshall Islands | 0.54(0.13 to 1.14) | 0.45(0.08 to 1.05) | 0.63(0.12 to 1.35) | 11.03(2.73 to 23.15) | 9.25(1.68 to 22.11) | 12.65(2.51 to 27.51) |
| Mauritania | 0.15(0.03 to 0.37) | 0.1(0.02 to 0.25) | 0.2(0.03 to 0.52) | 2.42(0.48 to 5.92) | 1.74(0.25 to 4.31) | 3.07(0.46 to 8.27) |
| Mauritius | 0.36(0.08 to 0.78) | 0.17(0.03 to 0.39) | 0.64(0.12 to 1.39) | 6.69(1.43 to 14.35) | 3.14(0.61 to 7.12) | 11.29(2.1 to 24.86) |
| Mexico | 0.23(0.05 to 0.47) | 0.14(0.03 to 0.31) | 0.33(0.06 to 0.72) | 4.09(0.93 to 8.34) | 2.43(0.46 to 5.42) | 6.03(1.01 to 12.9) |
| Micronesia (Federated States of) | 0.4(0.09 to 0.86) | 0.34(0.06 to 0.84) | 0.48(0.09 to 1.09) | 8.05(1.84 to 17.84) | 6.85(1.15 to 17.26) | 9.53(1.68 to 22.33) |
| Monaco | 0.86(0.16 to 2.26) | 0.35(0.05 to 1) | 1.51(0.24 to 3.84) | 14.46(2.63 to 38.34) | 5.61(0.79 to 16.33) | 24.95(3.9 to 62.89) |
| Mongolia | 0.06(0.01 to 0.14) | 0.03(0.01 to 0.08) | 0.1(0.01 to 0.24) | 1.02(0.2 to 2.49) | 0.58(0.09 to 1.55) | 1.69(0.26 to 4.29) |
| Montenegro | 0.56(0.12 to 1.22) | 0.25(0.05 to 0.58) | 0.99(0.18 to 2.22) | 10.38(2.12 to 22.73) | 4.54(0.84 to 10.56) | 17.92(3.13 to 40.26) |
| Morocco | 0.3(0.06 to 0.67) | 0.09(0.01 to 0.2) | 0.52(0.09 to 1.2) | 5.34(1.03 to 11.94) | 1.5(0.26 to 3.52) | 9.34(1.52 to 21.45) |
| Mozambique | 0.23(0.05 to 0.53) | 0.12(0.02 to 0.31) | 0.4(0.07 to 0.97) | 4.1(0.87 to 9.68) | 2.07(0.33 to 5.56) | 7.04(1.16 to 16.7) |
| Myanmar | 0.2(0.04 to 0.44) | 0.12(0.02 to 0.27) | 0.32(0.06 to 0.76) | 3.37(0.7 to 7.49) | 1.97(0.36 to 4.58) | 5.36(0.93 to 12.98) |
| Namibia | 0.19(0.04 to 0.42) | 0.13(0.02 to 0.32) | 0.27(0.04 to 0.64) | 3.31(0.71 to 7.51) | 2.25(0.36 to 5.69) | 4.88(0.81 to 11.49) |
| Nauru | 0.4(0.09 to 0.85) | 0.3(0.05 to 0.72) | 0.49(0.09 to 1.12) | 7.97(1.77 to 17.41) | 6.15(1.02 to 15.1) | 9.98(1.71 to 23.95) |
| Nepal | 0.14(0.03 to 0.33) | 0.09(0.01 to 0.22) | 0.2(0.03 to 0.49) | 2.35(0.49 to 5.56) | 1.41(0.23 to 3.61) | 3.39(0.58 to 8.48) |
| Netherlands | 0.43(0.08 to 0.97) | 0.19(0.03 to 0.43) | 0.78(0.13 to 1.8) | 7.21(1.38 to 16.31) | 3.07(0.54 to 7.27) | 12.32(1.95 to 28.54) |
| New Zealand | 0.24(0.05 to 0.53) | 0.12(0.02 to 0.28) | 0.39(0.06 to 0.91) | 3.68(0.73 to 8.18) | 1.81(0.31 to 4.31) | 5.89(0.94 to 13.73) |
| Nicaragua | 0.16(0.03 to 0.33) | 0.11(0.02 to 0.25) | 0.22(0.04 to 0.5) | 2.65(0.58 to 5.64) | 1.8(0.33 to 4.13) | 3.75(0.62 to 8.56) |
| Niger | 0.13(0.02 to 0.35) | 0.07(0.01 to 0.2) | 0.2(0.03 to 0.57) | 2.23(0.43 to 5.77) | 1.26(0.15 to 3.49) | 3.27(0.47 to 9.29) |
| Nigeria | 0.08(0.02 to 0.19) | 0.05(0.01 to 0.13) | 0.12(0.02 to 0.28) | 1.29(0.26 to 3.03) | 0.8(0.13 to 2.01) | 1.79(0.31 to 4.38) |
| Niue | 0.4(0.1 to 0.82) | 0.3(0.06 to 0.68) | 0.55(0.11 to 1.16) | 8.08(1.97 to 16.71) | 5.74(1.07 to 13.08) | 10.9(2.12 to 23.14) |
| North Macedonia | 0.78(0.16 to 1.71) | 0.26(0.05 to 0.61) | 1.41(0.26 to 3.14) | 14.97(3.05 to 33.58) | 4.57(0.86 to 10.86) | 26.85(4.77 to 60.45) |
| Northern Mariana Islands | 0.37(0.09 to 0.79) | 0.44(0.08 to 0.99) | 0.29(0.05 to 0.65) | 6.86(1.6 to 14.45) | 8.07(1.44 to 18.3) | 5.61(1 to 12.57) |
| Norway | 0.44(0.09 to 0.98) | 0.21(0.04 to 0.49) | 0.76(0.13 to 1.71) | 7.2(1.48 to 16.16) | 3.39(0.61 to 7.94) | 11.8(1.92 to 27.03) |
| Oman | 0.35(0.08 to 0.75) | 0.21(0.04 to 0.48) | 0.5(0.09 to 1.12) | 6.17(1.32 to 13.3) | 3.66(0.66 to 8.26) | 8.53(1.53 to 19.39) |
| Pakistan | 0.65(0.13 to 1.48) | 0.31(0.05 to 0.75) | 0.98(0.16 to 2.28) | 12.14(2.41 to 28.01) | 5.76(0.98 to 13.79) | 18.17(3.08 to 42.71) |
| Palau | 0.17(0.04 to 0.36) | 0.1(0.02 to 0.21) | 0.25(0.05 to 0.54) | 3.31(0.75 to 7.2) | 1.74(0.31 to 3.89) | 4.95(0.96 to 11.01) |
| Palestine | 0.57(0.12 to 1.21) | 0.18(0.03 to 0.41) | 1.13(0.21 to 2.44) | 10.4(2.27 to 22.05) | 3.15(0.58 to 7.11) | 19.25(3.63 to 41.63) |
| Panama | 0.17(0.04 to 0.39) | 0.11(0.02 to 0.25) | 0.25(0.04 to 0.55) | 2.94(0.65 to 6.62) | 1.84(0.33 to 4.37) | 4.14(0.71 to 9.33) |
| Papua New Guinea | 0.21(0.05 to 0.5) | 0.14(0.02 to 0.38) | 0.27(0.04 to 0.64) | 4.19(0.87 to 9.92) | 2.87(0.47 to 8.02) | 5.38(0.9 to 12.75) |
| Paraguay | 0.14(0.03 to 0.32) | 0.06(0.01 to 0.15) | 0.24(0.04 to 0.55) | 2.47(0.49 to 5.7) | 1.03(0.18 to 2.56) | 4.12(0.67 to 9.66) |
| Peru | 0.11(0.02 to 0.25) | 0.07(0.01 to 0.19) | 0.14(0.02 to 0.35) | 1.67(0.34 to 4.05) | 1.16(0.2 to 2.97) | 2.23(0.37 to 5.49) |
| Philippines | 0.09(0.02 to 0.21) | 0.06(0.01 to 0.14) | 0.14(0.02 to 0.33) | 1.65(0.34 to 3.7) | 1.01(0.18 to 2.34) | 2.48(0.38 to 6.01) |
| Poland | 0.68(0.13 to 1.53) | 0.21(0.04 to 0.49) | 1.46(0.24 to 3.38) | 12.88(2.48 to 29.21) | 3.64(0.64 to 8.49) | 26.04(4.3 to 60.07) |
| Portugal | 0.57(0.12 to 1.22) | 0.23(0.04 to 0.52) | 1.07(0.18 to 2.34) | 9.86(1.92 to 20.94) | 3.47(0.64 to 7.78) | 18.24(3.16 to 40.12) |
| Puerto Rico | 0.37(0.09 to 0.81) | 0.2(0.04 to 0.46) | 0.62(0.11 to 1.35) | 6.22(1.39 to 13.76) | 3.17(0.6 to 7.56) | 10.15(1.89 to 22.47) |
| Qatar | 1.4(0.36 to 2.86) | 1.27(0.27 to 2.76) | 1.43(0.32 to 3.05) | 19.04(4.73 to 39.39) | 16.65(3.62 to 35.56) | 19.74(4.31 to 42.21) |
| Republic of Korea | 0.23(0.04 to 0.5) | 0.08(0.01 to 0.19) | 0.48(0.08 to 1.11) | 3.63(0.69 to 8.06) | 1.17(0.21 to 2.76) | 7.19(1.19 to 16.75) |
| Republic of Moldova | 0.22(0.04 to 0.5) | 0.06(0.01 to 0.16) | 0.45(0.07 to 1.07) | 4.62(0.78 to 10.81) | 1.21(0.2 to 2.95) | 9.59(1.53 to 22.94) |
| Romania | 0.37(0.07 to 0.85) | 0.12(0.02 to 0.28) | 0.75(0.12 to 1.75) | 7.46(1.37 to 17.07) | 2.08(0.34 to 4.98) | 14.63(2.46 to 34.53) |
| Russian Federation | 0.17(0.03 to 0.38) | 0.06(0.01 to 0.14) | 0.39(0.06 to 0.93) | 3.2(0.61 to 7.45) | 1.03(0.18 to 2.55) | 7.13(1.11 to 17.19) |
| Rwanda | 0.17(0.04 to 0.39) | 0.11(0.02 to 0.28) | 0.26(0.04 to 0.67) | 2.85(0.6 to 6.78) | 1.85(0.31 to 4.74) | 4.42(0.67 to 11.43) |
| Saint Kitts and Nevis | 0.52(0.12 to 1.12) | 0.38(0.07 to 0.88) | 0.71(0.12 to 1.57) | 9.19(2.12 to 19.75) | 6.35(1.19 to 14.85) | 12.46(2.16 to 27.95) |
| Saint Lucia | 0.59(0.14 to 1.21) | 0.41(0.08 to 0.89) | 0.83(0.16 to 1.77) | 9.98(2.36 to 20.7) | 6.87(1.33 to 15.23) | 13.72(2.67 to 29.24) |
| Saint Vincent and the Grenadines | 0.45(0.1 to 0.92) | 0.25(0.05 to 0.54) | 0.66(0.13 to 1.4) | 7.66(1.71 to 16.05) | 4.18(0.8 to 9.3) | 11.09(2.11 to 23.95) |
| Samoa | 0.32(0.08 to 0.69) | 0.25(0.05 to 0.61) | 0.4(0.07 to 0.9) | 6.11(1.44 to 13.55) | 4.98(0.87 to 12.08) | 7.43(1.3 to 16.76) |
| San Marino | 0.8(0.14 to 2.05) | 0.29(0.05 to 0.81) | 1.46(0.23 to 3.65) | 13.19(2.34 to 33.51) | 4.52(0.71 to 12.78) | 23.25(3.69 to 58.61) |
| Sao Tome and Principe | 0.49(0.1 to 1.1) | 0.23(0.03 to 0.56) | 0.81(0.14 to 1.91) | 7.88(1.55 to 17.77) | 3.56(0.54 to 8.66) | 12.79(2.19 to 31) |
| Saudi Arabia | 0.27(0.06 to 0.57) | 0.13(0.02 to 0.3) | 0.37(0.07 to 0.79) | 4.83(1.06 to 10.45) | 2.24(0.41 to 5.23) | 6.59(1.24 to 14.67) |
| Senegal | 0.32(0.06 to 0.74) | 0.15(0.02 to 0.37) | 0.51(0.08 to 1.23) | 5.33(1.03 to 12.58) | 2.58(0.4 to 6.31) | 8.38(1.4 to 20.28) |
| Serbia | 0.74(0.14 to 1.63) | 0.29(0.05 to 0.67) | 1.33(0.23 to 3) | 13.67(2.7 to 30.25) | 4.93(0.91 to 11.75) | 24.43(4.2 to 55.63) |
| Seychelles | 0.86(0.21 to 1.76) | 0.51(0.1 to 1.12) | 1.36(0.25 to 2.88) | 13.85(3.22 to 28.68) | 7.53(1.5 to 16.51) | 21.87(3.89 to 46.86) |
| Sierra Leone | 0.13(0.03 to 0.32) | 0.08(0.01 to 0.21) | 0.19(0.03 to 0.48) | 2.22(0.41 to 5.35) | 1.44(0.18 to 3.68) | 3.04(0.48 to 7.88) |
| Singapore | 0.15(0.03 to 0.35) | 0.07(0.01 to 0.16) | 0.27(0.05 to 0.63) | 2.58(0.5 to 5.89) | 1.06(0.19 to 2.54) | 4.4(0.74 to 10.17) |
| Slovakia | 0.39(0.08 to 0.89) | 0.13(0.02 to 0.34) | 0.83(0.14 to 1.94) | 7.31(1.41 to 16.73) | 2.34(0.4 to 5.95) | 14.68(2.45 to 34.39) |
| Slovenia | 0.41(0.08 to 0.96) | 0.13(0.02 to 0.34) | 0.86(0.14 to 2.05) | 6.96(1.26 to 16.1) | 2.24(0.38 to 5.69) | 13.48(2.12 to 32.03) |
| Solomon Islands | 0.33(0.07 to 0.73) | 0.24(0.04 to 0.63) | 0.41(0.07 to 0.97) | 7.08(1.53 to 16.15) | 5.32(0.88 to 14.73) | 8.75(1.43 to 21.17) |
| Somalia | 0.15(0.03 to 0.45) | 0.11(0.02 to 0.35) | 0.23(0.03 to 0.67) | 2.89(0.53 to 8.5) | 2.12(0.28 to 6.71) | 4.14(0.56 to 11.95) |
| South Africa | 0.24(0.05 to 0.51) | 0.12(0.02 to 0.27) | 0.45(0.08 to 1.02) | 4.13(0.86 to 8.91) | 2.08(0.38 to 4.66) | 7.42(1.28 to 16.81) |
| South Sudan | 0.19(0.03 to 0.5) | 0.1(0.01 to 0.28) | 0.27(0.04 to 0.73) | 3.25(0.6 to 8.87) | 1.77(0.26 to 4.87) | 4.53(0.7 to 12.72) |
| Spain | 0.72(0.15 to 1.57) | 0.2(0.04 to 0.45) | 1.46(0.25 to 3.29) | 12.39(2.45 to 27.27) | 3.01(0.55 to 6.88) | 24.37(4.17 to 54.66) |
| Sri Lanka | 0.24(0.05 to 0.54) | 0.11(0.02 to 0.25) | 0.44(0.08 to 0.97) | 3.97(0.85 to 8.95) | 1.7(0.34 to 3.91) | 7.09(1.29 to 15.91) |
| Sudan | 0.42(0.08 to 1.16) | 0.19(0.03 to 0.57) | 0.61(0.1 to 1.69) | 7.39(1.38 to 19.59) | 3.25(0.54 to 9.71) | 10.77(1.69 to 29.43) |
| Suriname | 0.3(0.07 to 0.63) | 0.16(0.03 to 0.36) | 0.49(0.09 to 1.06) | 5.49(1.24 to 11.84) | 2.86(0.55 to 6.56) | 8.75(1.67 to 19.36) |
| Sweden | 0.38(0.08 to 0.85) | 0.18(0.03 to 0.41) | 0.65(0.11 to 1.48) | 6.35(1.24 to 13.94) | 2.86(0.52 to 6.8) | 10.39(1.72 to 23.62) |
| Switzerland | 0.34(0.07 to 0.78) | 0.15(0.03 to 0.35) | 0.61(0.1 to 1.41) | 5.67(1.11 to 12.7) | 2.43(0.44 to 5.73) | 9.67(1.64 to 22.19) |
| Syrian Arab Republic | 0.26(0.05 to 0.59) | 0.11(0.02 to 0.25) | 0.39(0.06 to 0.91) | 4.52(0.87 to 10.53) | 1.64(0.29 to 3.85) | 7.16(1.18 to 17.15) |
| Taiwan (Province of China) | 0.37(0.08 to 0.82) | 0.23(0.04 to 0.55) | 0.54(0.09 to 1.28) | 6.3(1.26 to 14.27) | 3.64(0.61 to 9.05) | 9.4(1.55 to 22.43) |
| Tajikistan | 0.15(0.03 to 0.34) | 0.08(0.01 to 0.2) | 0.24(0.04 to 0.58) | 2.68(0.55 to 6.21) | 1.48(0.26 to 3.54) | 4.11(0.64 to 9.77) |
| Thailand | 0.17(0.04 to 0.39) | 0.08(0.01 to 0.19) | 0.29(0.05 to 0.68) | 2.64(0.53 to 6.07) | 1.1(0.19 to 2.73) | 4.59(0.74 to 10.74) |
| Timor-Leste | 0.18(0.04 to 0.4) | 0.1(0.02 to 0.23) | 0.26(0.04 to 0.63) | 2.85(0.57 to 6.54) | 1.55(0.25 to 3.83) | 4.18(0.71 to 10.33) |
| Togo | 0.15(0.03 to 0.37) | 0.08(0.01 to 0.2) | 0.28(0.04 to 0.71) | 2.66(0.5 to 6.48) | 1.36(0.17 to 3.39) | 4.74(0.71 to 12.07) |
| Tokelau | 0.27(0.07 to 0.61) | 0.27(0.05 to 0.64) | 0.28(0.05 to 0.63) | 5.34(1.29 to 12.1) | 5.48(0.96 to 13.11) | 5.23(0.9 to 12.28) |
| Tonga | 0.25(0.06 to 0.56) | 0.16(0.03 to 0.39) | 0.36(0.06 to 0.86) | 4.84(1.07 to 11.03) | 3.1(0.59 to 7.47) | 6.89(1.1 to 16.68) |
| Trinidad and Tobago | 0.28(0.06 to 0.6) | 0.15(0.03 to 0.35) | 0.43(0.08 to 0.92) | 5.33(1.19 to 11.76) | 2.68(0.52 to 6.33) | 8.25(1.51 to 18.07) |
| Tunisia | 0.66(0.13 to 1.49) | 0.15(0.03 to 0.36) | 1.26(0.23 to 2.87) | 11.64(2.29 to 26.25) | 2.46(0.44 to 6.01) | 21.65(3.75 to 49.16) |
| Turkey | 0.37(0.07 to 0.84) | 0.12(0.02 to 0.28) | 0.68(0.11 to 1.58) | 7(1.26 to 15.74) | 1.96(0.34 to 4.64) | 12.9(2.08 to 30.13) |
| Turkmenistan | 0.1(0.02 to 0.23) | 0.05(0.01 to 0.12) | 0.17(0.03 to 0.42) | 2.11(0.39 to 4.88) | 0.94(0.15 to 2.36) | 3.62(0.55 to 8.79) |
| Tuvalu | 0.32(0.08 to 0.71) | 0.28(0.05 to 0.68) | 0.37(0.07 to 0.85) | 6.31(1.48 to 14.3) | 5.51(0.97 to 13.42) | 7.23(1.29 to 16.73) |
| Uganda | 0.23(0.05 to 0.53) | 0.16(0.03 to 0.4) | 0.34(0.06 to 0.83) | 4.07(0.87 to 9.31) | 2.81(0.45 to 6.83) | 5.86(0.98 to 14.31) |
| Ukraine | 0.18(0.03 to 0.43) | 0.04(0.01 to 0.1) | 0.44(0.07 to 1.06) | 3.94(0.72 to 9.26) | 0.86(0.14 to 2.04) | 9.11(1.36 to 21.79) |
| United Arab Emirates | 1.06(0.18 to 2.83) | 0.46(0.06 to 1.27) | 1.32(0.21 to 3.58) | 19.64(3.29 to 53.28) | 8.03(0.96 to 22.27) | 24.54(3.91 to 66.02) |
| United Kingdom | 0.68(0.15 to 1.43) | 0.36(0.07 to 0.8) | 1.12(0.2 to 2.43) | 10.74(2.39 to 22.57) | 5.56(1.05 to 12.39) | 17.03(3.02 to 37.03) |
| United Republic of Tanzania | 0.17(0.04 to 0.41) | 0.11(0.02 to 0.27) | 0.24(0.04 to 0.64) | 2.95(0.59 to 7.35) | 1.93(0.31 to 4.77) | 4.14(0.64 to 11.05) |
| United States of America | 0.53(0.11 to 1.12) | 0.22(0.04 to 0.48) | 0.96(0.17 to 2.06) | 9.04(1.92 to 18.95) | 3.63(0.69 to 8.16) | 15.76(2.87 to 33.86) |
| United States Virgin Islands | 0.3(0.06 to 0.65) | 0.1(0.02 to 0.23) | 0.6(0.1 to 1.32) | 5.46(1.13 to 11.85) | 1.62(0.3 to 3.7) | 10.49(1.88 to 23.39) |
| Uruguay | 0.41(0.08 to 0.92) | 0.12(0.02 to 0.29) | 0.84(0.13 to 1.96) | 7.23(1.31 to 16.47) | 1.95(0.32 to 4.56) | 14.6(2.26 to 33.9) |
| Uzbekistan | 0.18(0.04 to 0.4) | 0.08(0.01 to 0.18) | 0.33(0.05 to 0.77) | 3.65(0.73 to 8.32) | 1.33(0.22 to 3.1) | 6.71(1.12 to 15.85) |
| Vanuatu | 0.28(0.06 to 0.65) | 0.22(0.04 to 0.6) | 0.34(0.06 to 0.78) | 5.59(1.17 to 13.09) | 4.39(0.69 to 12.13) | 6.67(1.17 to 15.67) |
| Venezuela (Bolivarian Republic of) | 0.24(0.05 to 0.55) | 0.15(0.03 to 0.35) | 0.36(0.06 to 0.83) | 4.42(0.95 to 10.19) | 2.59(0.47 to 6.31) | 6.58(1.1 to 15.13) |
| Viet Nam | 0.19(0.04 to 0.4) | 0.08(0.01 to 0.2) | 0.37(0.06 to 0.83) | 3.13(0.65 to 6.95) | 1.26(0.22 to 2.96) | 6.01(0.97 to 13.94) |
| Yemen | 0.27(0.05 to 0.66) | 0.13(0.02 to 0.35) | 0.42(0.07 to 1.06) | 4.77(0.96 to 11.74) | 2.31(0.38 to 6.25) | 7.34(1.23 to 18.7) |
| Zambia | 0.23(0.04 to 0.52) | 0.14(0.02 to 0.36) | 0.34(0.06 to 0.83) | 4.04(0.77 to 9.33) | 2.46(0.38 to 6.37) | 5.94(1.03 to 14.71) |
| Zimbabwe | 0.92(0.19 to 2.09) | 0.88(0.12 to 2.15) | 1(0.15 to 2.4) | 16.75(3.33 to 38.09) | 16.08(2.24 to 40.38) | 17.88(2.69 to 43.16) |

Abbreviations: FPG fasting plasma glucose; DALY disability-adjusted life year; UI uncertainty interval; ASDR age-standardized death rate.

Table S2 **Percentage change in bladder cancer burden attributable to high FPG among 204 countries and territories from 1990 to 2019.**

| location | Percentage change in ASDR, 1990 to 2019 | | | Percentage change in age-standardized DALY rate, 1990 to 2019 | | |
| --- | --- | --- | --- | --- | --- | --- |
|  | Both | Female | Male | Both | Female | Male |
| Afghanistan | 71.39(57.28 to 97.87) | 76.69(62.36 to 101.05) | 68.8(55.65 to 90.91) | 73.49(58.54 to 99.02) | 80(64.7 to 105.83) | 70.78(56.51 to 93.92) |
| Albania | 47.38(36.78 to 62.58) | 48.05(37.4 to 63.72) | 44.63(31.77 to 61.54) | 49.88(38.87 to 65.08) | 54.06(41.17 to 72.3) | 44.98(32.15 to 61.96) |
| Algeria | 64.35(51.9 to 88.85) | 70.72(57.24 to 95.54) | 63.58(51.07 to 86.24) | 68.23(54.94 to 91.73) | 76.87(61.47 to 103.22) | 67.16(54.07 to 90.42) |
| American Samoa | 32.15(18.19 to 61.45) | 40.26(28.81 to 59.94) | 28.62(19.77 to 44.7) | 37.43(23.46 to 68.83) | 42.26(30.1 to 62.26) | 35.06(24.91 to 53.38) |
| Andorra | 57.83(46.79 to 77.25) | 59.99(48.27 to 78.3) | 58.11(46.61 to 77.34) | 59.78(48.23 to 78.65) | 63.94(51.66 to 83.12) | 59.6(47.73 to 78.96) |
| Angola | 27.55(17.61 to 40.52) | 25.52(16.18 to 37.07) | 28.4(18.55 to 41.29) | 31.52(20.51 to 45.8) | 30.41(19.62 to 45.27) | 32.1(20.75 to 47.47) |
| Antigua and Barbuda | 39.08(28.98 to 55.01) | 38.08(27.27 to 54.62) | 39.33(28.81 to 56.47) | 43.22(31.33 to 62.38) | 44.38(32.61 to 62.85) | 42.52(30.93 to 61.27) |
| Argentina | 56.64(44.87 to 75.77) | 36.93(24.43 to 55.49) | 63.05(49.04 to 85.77) | 58.32(46.16 to 77.2) | 37.52(24.96 to 55.42) | 63.95(49.97 to 86.36) |
| Armenia | 74.47(58.93 to 97.72) | 60.7(47.79 to 81.84) | 76.56(58.52 to 101.91) | 76.57(60.98 to 99.85) | 62.86(50.01 to 84.66) | 78.04(60.03 to 102.8) |
| Australia | 50.9(38.03 to 70.49) | 52.59(36.36 to 76.89) | 42.64(28.47 to 64.97) | 62.23(49.41 to 82.43) | 61.9(45.4 to 87.47) | 54.93(40.42 to 78.03) |
| Austria | 80.57(64.34 to 108.16) | 66.47(53.06 to 89.29) | 84.35(66.76 to 111.27) | 82.66(67.59 to 108.78) | 67.85(54.09 to 90.46) | 84.5(67.41 to 111.86) |
| Azerbaijan | 97.15(79.19 to 126.71) | 98(76.18 to 132.15) | 93.55(74.43 to 122.16) | 100.52(81.41 to 130.86) | 104.09(80.29 to 140.5) | 96.54(76.13 to 128.15) |
| Bahamas | 35.3(26.32 to 49.55) | 34.65(24.58 to 51.1) | 33.88(22.98 to 50.49) | 37.34(27.93 to 52.37) | 35.81(24.54 to 52.73) | 36.69(25.27 to 54.28) |
| Bahrain | 48.01(33.73 to 79.27) | 58.9(43.25 to 85.89) | 47.17(33.64 to 70.84) | 50.54(35.49 to 81.77) | 63.63(46.72 to 93.78) | 48.96(34.33 to 73.88) |
| Bangladesh | 36.98(26.78 to 50) | 49.86(38.41 to 66.98) | 32.75(22.47 to 45.54) | 42.35(31.29 to 57.05) | 55.44(42.19 to 75.42) | 37.64(26.75 to 51.53) |
| Barbados | 30.56(22.59 to 44.71) | 22.12(13.47 to 35.37) | 35.87(24.83 to 53.75) | 34.39(25.4 to 48.33) | 26.6(17.01 to 41.07) | 38.49(26.71 to 55.82) |
| Belarus | 24.18(12.57 to 38.69) | 13.67(1.17 to 27.23) | 26.24(13.09 to 42.21) | 26.02(13.81 to 41.27) | 12.73(-0.86 to 26.73) | 27.64(14.13 to 43.62) |
| Belgium | 52.52(39.83 to 72.75) | 42.38(29.63 to 61.45) | 54.69(40.68 to 76.68) | 54.03(41.68 to 73.2) | 42.26(29.18 to 62.18) | 55.52(41.68 to 77.4) |
| Belize | 48.48(35.87 to 67.99) | 46.73(35.78 to 64.92) | 52.89(39.71 to 73.77) | 51.08(38.24 to 70.61) | 48.33(36.83 to 67.59) | 56.03(42.24 to 77.36) |
| Benin | 47.28(32.36 to 67.94) | 55.73(42.61 to 74.05) | 46.48(33.68 to 64.5) | 49.33(33.18 to 71.29) | 61.84(46.14 to 83.18) | 48.06(32.83 to 67.59) |
| Bermuda | 34.31(25.03 to 47.79) | 38.73(26.28 to 54.47) | 29.83(19.43 to 44.95) | 36.64(26.04 to 51.21) | 38.49(25.99 to 53.67) | 33.18(22.19 to 48.19) |
| Bhutan | 68.63(50.68 to 98.39) | 63.97(51.57 to 83.75) | 69.34(57.56 to 88.77) | 76.09(57.81 to 105.43) | 71.37(56.63 to 95.52) | 75.12(61.46 to 97.47) |
| Bolivia (Plurinational State of) | 44.54(35.33 to 59.77) | 41.52(30.88 to 57.85) | 45.24(34.29 to 63) | 50.06(39.35 to 67.36) | 45.68(33.25 to 63.91) | 51.8(39.31 to 71.94) |
| Bosnia and Herzegovina | 87.91(72.02 to 114.94) | 74.62(58.19 to 103.8) | 90.11(71.63 to 123.39) | 96.41(79.55 to 125.28) | 76.83(60.53 to 105.92) | 99.36(79.57 to 134.28) |
| Botswana | 52.92(41.56 to 72.09) | 52.09(38.41 to 72.22) | 52.87(40.54 to 72.94) | 58.17(44.45 to 80.38) | 60.49(44.23 to 85.75) | 57.63(42.8 to 81.68) |
| Brazil | 11.88(7.32 to 18.76) | 11.21(7.05 to 16.07) | 12.55(8.54 to 19.08) | 12.77(8.34 to 19.71) | 10.54(6.3 to 15.3) | 14.56(10.4 to 21.59) |
| Brunei Darussalam | 7.06(0.11 to 15.59) | 7.51(0.01 to 17.45) | 8.79(4.14 to 16.76) | 7.33(0.11 to 16.36) | 9.14(1.06 to 19.82) | 8.79(4.06 to 16.38) |
| Bulgaria | 41.93(30.89 to 58.84) | 32.64(21.18 to 50.75) | 44.72(31.4 to 65.13) | 45.44(33.5 to 61.77) | 33.36(21.35 to 52.09) | 48.92(35.06 to 69.5) |
| Burkina Faso | 33.67(17.23 to 56.32) | 30.86(20.29 to 45.45) | 35.33(23.83 to 50.04) | 35.14(16.07 to 58.36) | 34.37(22.17 to 50.57) | 36.46(23.92 to 53.95) |
| Burundi | 23.62(6.52 to 44.02) | 20.76(10.82 to 32.13) | 22.54(11.81 to 34.99) | 28.75(10.5 to 50.66) | 25.99(14.53 to 39.88) | 27.02(15.19 to 41.71) |
| Cabo Verde | 60.37(45.15 to 84.13) | 79.34(63.06 to 106.23) | 58.25(42.32 to 81.17) | 61.62(43.59 to 87.94) | 90.51(70.86 to 121.44) | 62.76(43.88 to 90) |
| Cambodia | 130.66(108.63 to 169.27) | 127.61(105.23 to 166.06) | 132.84(108.76 to 175.08) | 135.75(111.96 to 175.84) | 133.65(108.6 to 176.31) | 138.13(111.97 to 182.69) |
| Cameroon | 52.72(39.31 to 71.81) | 61.15(48.92 to 79.44) | 50.44(37.2 to 70.67) | 55.13(40.62 to 75.43) | 68.44(53.37 to 91.02) | 51.81(35.72 to 73.8) |
| Canada | 60.55(46.4 to 80.55) | 55.62(38.75 to 76.52) | 59.31(43.86 to 81.26) | 68.1(52.91 to 88.68) | 60.37(43.18 to 80.68) | 67.06(50.12 to 89.34) |
| Central African Republic | 31.16(21.18 to 44.05) | 34.28(24.69 to 47.47) | 31.48(22.45 to 44.97) | 34.67(23.65 to 48.5) | 39.08(27.99 to 53.82) | 34.97(24.64 to 49.49) |
| Chad | 41.95(30.38 to 59.2) | 52.46(40.3 to 70.89) | 37.25(24.83 to 53.14) | 44.14(32.3 to 62.46) | 58.85(44.69 to 80.38) | 38.4(25.17 to 54.86) |
| Chile | 61.64(48.67 to 84.25) | 53.09(37.69 to 77.78) | 66.11(48.57 to 95.9) | 63.23(49.48 to 84.95) | 53.66(37.18 to 78.68) | 67.95(50.48 to 98.91) |
| China | 14.95(1.48 to 27.69) | 7.68(1.7 to 14.17) | 15.85(9.81 to 23.02) | 18.83(4.38 to 32.25) | 12.99(6.37 to 20.44) | 18.9(12.27 to 26.84) |
| Colombia | 7.05(-2.82 to 17.16) | 2.73(-7.36 to 14.21) | 10.06(-2.05 to 22.99) | 10.86(0.95 to 21.7) | 4.74(-5.49 to 16.31) | 14.93(2.64 to 28.49) |
| Comoros | 22.32(10.19 to 35.26) | 18.74(6.59 to 32.34) | 25.74(12.36 to 41.39) | 27.97(11.89 to 44.04) | 24.57(6.26 to 41.67) | 31.57(14.7 to 51.26) |
| Congo | 28.55(19.08 to 43.13) | 36.24(25.8 to 50.56) | 24.88(15.42 to 38.22) | 32.15(21.27 to 48.49) | 41.7(29.71 to 58.67) | 28.02(17.46 to 44.46) |
| Cook Islands | 47.36(37.01 to 65.81) | 39.46(29.14 to 56.31) | 53.57(41.25 to 75.31) | 54.67(43.03 to 76.73) | 45.29(33.24 to 65.38) | 61.79(46.98 to 87.21) |
| Costa Rica | 21.87(10.57 to 37.44) | 18.81(8.6 to 32.37) | 23.14(10.02 to 40.87) | 27.01(15.43 to 43.32) | 24.06(12.72 to 37.85) | 28.2(14.96 to 46.68) |
| Croatia | 40.9(31.99 to 58.55) | 35.4(21.22 to 53.99) | 40.82(30.62 to 61.41) | 45.2(35.23 to 63.88) | 37.28(23.51 to 55.42) | 44.7(33.92 to 66.22) |
| Cuba | 22.27(13.64 to 34.59) | 16.56(4.43 to 30.94) | 25.3(15.78 to 41.04) | 25.99(16.81 to 39.94) | 18.7(6.97 to 34.07) | 29.46(19.2 to 46.58) |
| Cyprus | 6.81(-3.61 to 18.77) | -2.28(-11.41 to 6.78) | 7.49(-2.02 to 19.04) | 8.93(-2.87 to 21.88) | 1.77(-8.53 to 12.2) | 9.09(-1.08 to 22.03) |
| Czechia | 63.25(49.26 to 86.82) | 58.27(43.19 to 84.18) | 62.86(46.67 to 91.19) | 68.91(53.52 to 94.81) | 61.63(46.23 to 87.72) | 69.5(52.25 to 98.7) |
| Côte d'Ivoire | 45.22(32.25 to 62.7) | 58.48(46.38 to 78.91) | 41.41(28.71 to 59.37) | 46.53(32.1 to 66.58) | 65.32(50.33 to 87.87) | 42.24(28.19 to 62.37) |
| Democratic People's Republic of Korea | 45.77(34.72 to 64.31) | 45.42(34.73 to 60.8) | 45.42(35.18 to 61.2) | 48.96(35.48 to 69.05) | 48.21(34.61 to 66.14) | 48.52(36.19 to 65.69) |
| Democratic Republic of the Congo | 23.76(12.69 to 39.67) | 31.54(21.85 to 45.4) | 28.28(19.25 to 41.21) | 27.73(16.53 to 43.39) | 36.64(24.76 to 53.58) | 31.91(21.96 to 46.28) |
| Denmark | 89.56(72.99 to 115.94) | 96.9(76.92 to 128.05) | 85.68(65.16 to 116.18) | 91.12(74.54 to 117.34) | 100.07(79.73 to 131.46) | 87.3(67.19 to 116.54) |
| Djibouti | 34.31(21.96 to 53.05) | 24.76(15.11 to 38.42) | 34.84(23.59 to 51.53) | 41.02(27.27 to 61.28) | 31.57(19.31 to 48.4) | 41.59(28.23 to 59.2) |
| Dominica | 49.02(36.52 to 73.89) | 37.39(27.24 to 54.81) | 56.02(43.02 to 80.58) | 55.11(41.11 to 83.23) | 41.94(30.81 to 59.8) | 61.73(47.41 to 88.78) |
| Dominican Republic | 60.09(47.8 to 78.81) | 60.35(46.19 to 81.29) | 60.06(45.31 to 82.19) | 67.98(55.19 to 87.68) | 66.1(51.51 to 86.89) | 69.34(53.94 to 91.71) |
| Ecuador | 59.62(47.73 to 78.89) | 56.59(43.1 to 79.6) | 61.82(46.06 to 85.27) | 66.8(54.33 to 87.06) | 61.78(47.32 to 84.45) | 70.08(52.99 to 94.53) |
| Egypt | 142.87(121.6 to 182.39) | 98.14(80.28 to 128.23) | 155.77(133.48 to 194.11) | 150.82(128.54 to 189.56) | 112.49(92.04 to 148.77) | 160.15(137.45 to 199.82) |
| El Salvador | 57.33(44.96 to 79.42) | 64.19(48.21 to 88.75) | 56.45(42.39 to 78.15) | 66.26(52.94 to 89.53) | 73.55(56.58 to 99.91) | 65.48(51.29 to 88.69) |
| Equatorial Guinea | 44.15(30.14 to 66.56) | 52.36(39.51 to 70.14) | 39.52(27.95 to 56.74) | 48.93(33.05 to 74.4) | 60.33(44.21 to 82.65) | 43.72(29.9 to 64) |
| Eritrea | 35.81(24.2 to 54.09) | 29.66(19.24 to 43.64) | 40.06(28.95 to 55.43) | 41.62(28.8 to 59.75) | 36.15(22.91 to 52.12) | 45.75(33.81 to 62.67) |
| Estonia | 51.65(36.19 to 71.88) | 40.68(28.73 to 56.97) | 52.65(40.22 to 70.16) | 57.1(41.29 to 76.92) | 44.35(32.2 to 60.83) | 57.85(44.72 to 76.67) |
| Eswatini | 39.54(29.39 to 56.09) | 38.94(28.29 to 55.96) | 37.89(26.76 to 55.13) | 47.09(33.23 to 66.76) | 48.93(34.16 to 70.33) | 45.45(30.34 to 66.77) |
| Ethiopia | 4.32(-4.97 to 15.56) | 8.39(0.44 to 16.63) | -1.02(-7.55 to 6.43) | 6.15(-4.13 to 19.17) | 10.43(-0.11 to 21.4) | 1.02(-6.55 to 10.19) |
| Fiji | 46.21(33.85 to 69.33) | 48.87(36.65 to 72.11) | 46.69(34.23 to 69.39) | 52.81(40.43 to 77.56) | 50.78(37.54 to 74.89) | 55.52(41.27 to 82.96) |
| Finland | 43.59(33.21 to 59.54) | 35.26(21.95 to 53.19) | 44.93(32.92 to 61.46) | 47.86(37.13 to 63.55) | 39.64(25.93 to 58.54) | 47.63(35.55 to 64.43) |
| France | 50.93(38.37 to 66.98) | 47.69(33.19 to 66.8) | 49.49(33.47 to 68.71) | 52.93(40.33 to 69.27) | 47.44(33.7 to 65.87) | 51.54(36.24 to 70) |
| Gabon | 33.13(22.79 to 48.09) | 38.83(28.32 to 54.3) | 27.8(19.08 to 40.31) | 38.92(26.43 to 56.49) | 46.13(33.23 to 65.23) | 33.27(22.51 to 48.65) |
| Gambia | 51.48(39.83 to 67.99) | 52.84(40.4 to 70.33) | 47.58(34.42 to 65.22) | 56.55(43.5 to 75.79) | 59.6(45.4 to 80.95) | 52.34(38.31 to 71.31) |
| Georgia | 111.35(90.11 to 145.69) | 91.25(73.91 to 123.22) | 112.45(89.6 to 150.5) | 117.75(95.94 to 153.75) | 96.09(78.5 to 128.3) | 118.16(95.41 to 157.55) |
| Germany | 39.85(26.45 to 61.22) | 27.51(12.89 to 44.99) | 43.46(29.55 to 68.8) | 41.45(28.53 to 63.57) | 26.73(12.44 to 44.49) | 43.99(29.81 to 69.66) |
| Ghana | 55.81(37.48 to 82.04) | 42.33(30.86 to 58.23) | 65.76(50.59 to 91.13) | 59.06(36.74 to 85.79) | 48.78(34.88 to 66.39) | 69.78(51 to 99.28) |
| Greece | 52.94(38.69 to 72.7) | 36.24(22.82 to 54.35) | 55.75(39.68 to 79.35) | 55.96(40.81 to 77.37) | 37.08(23.34 to 55.16) | 58.23(41.14 to 81.91) |
| Greenland | 109.02(90.31 to 138.69) | 146.17(124.66 to 176.48) | 91.29(74.22 to 119.19) | 107.4(89.18 to 137.22) | 141.16(118.64 to 170.61) | 89.88(72.38 to 117.96) |
| Grenada | 48.12(37.32 to 67.57) | 37.6(27.66 to 54.68) | 55.13(41.72 to 79.81) | 53.47(42.48 to 73.14) | 41.98(30.77 to 60.69) | 59.65(45.25 to 84.77) |
| Guam | 29.6(21.49 to 42.15) | 21.36(12.37 to 33.82) | 31.94(21.59 to 46.06) | 32.48(23.31 to 45.95) | 16.91(8.38 to 28.45) | 36.06(24.56 to 52.04) |
| Guatemala | 58.82(43.16 to 88.99) | 53.45(38.97 to 76.92) | 61.93(46.22 to 89.07) | 68.63(52.83 to 99.31) | 65.27(49.74 to 91.57) | 72.17(55.13 to 101.45) |
| Guinea | 52.96(41.2 to 70.87) | 60.37(47.63 to 79.1) | 51.27(38.45 to 70.59) | 58.58(44.89 to 77.76) | 66.2(51.53 to 87.75) | 56.38(42.1 to 76.79) |
| Guinea-Bissau | 42.27(29.89 to 60.13) | 51.83(39.94 to 70.3) | 40.17(28.17 to 57.7) | 42.55(29.16 to 61.27) | 57.68(43.92 to 77.83) | 39.5(25.97 to 58.62) |
| Guyana | 39.04(29.45 to 55.28) | 27.41(18.85 to 42.52) | 45.16(33.23 to 65.74) | 41.31(31.08 to 58.67) | 29.66(20 to 46.69) | 47.29(35.27 to 68.21) |
| Haiti | 43.54(34.58 to 60.67) | 50.77(39.54 to 68.84) | 36.47(26.38 to 50.62) | 46.44(36.69 to 65.15) | 52.02(39.45 to 71.57) | 40.12(29.05 to 55.9) |
| Honduras | 25.72(14.68 to 42.75) | 36.79(25.57 to 53.82) | 24.19(15.35 to 37.2) | 35(23.03 to 52.9) | 48.74(35.77 to 68.32) | 32.38(21.93 to 47.98) |
| Hungary | 46.17(35.78 to 64.03) | 33.78(20.68 to 53.6) | 50.93(37.3 to 71.47) | 50.37(38.96 to 68.75) | 35.85(21.6 to 56.46) | 55.72(42.48 to 76.93) |
| Iceland | 79.86(64.59 to 105.16) | 68.29(53.97 to 90.43) | 80.27(65.18 to 103.45) | 82.77(66.34 to 109.07) | 70.66(56.12 to 92.79) | 82.12(67.4 to 105.21) |
| India | 50.07(42.95 to 62.11) | 51.6(45.02 to 62.41) | 49.46(42.55 to 60.07) | 55.77(48.61 to 68.08) | 57.77(50.78 to 69.5) | 54.82(47.41 to 66.32) |
| Indonesia | 49.97(41.48 to 62.24) | 61.61(52.91 to 74.19) | 45.08(38.06 to 56.01) | 57.5(48.38 to 71.88) | 68.02(58.42 to 81.78) | 52.79(45.45 to 64.93) |
| Iran (Islamic Republic of) | 70.8(60.04 to 89.78) | 87.29(74.14 to 109.77) | 64.89(55.2 to 81.28) | 74.54(63.2 to 94.84) | 93.86(77.73 to 118.13) | 68.69(58.42 to 85.58) |
| Iraq | 49.85(36.74 to 72.05) | 56.46(41.34 to 82.35) | 48.19(34.78 to 69.35) | 51.7(37.72 to 74.63) | 57.25(41.77 to 83.31) | 50.17(36.3 to 71.98) |
| Ireland | 173.27(147.66 to 219.55) | 157.35(132.26 to 199.44) | 179.89(148.09 to 236) | 172.2(146.2 to 217.23) | 157.72(132.68 to 196.74) | 177.05(146.71 to 230.43) |
| Israel | 54.95(43.69 to 71.81) | 46.31(33.63 to 67.08) | 57.39(43.9 to 80.03) | 53.92(42.74 to 71.77) | 46.18(33.74 to 67.25) | 56.53(42.95 to 79.04) |
| Italy | 43.32(36.21 to 55.59) | 27.63(21.68 to 36.39) | 45.99(38.43 to 59.78) | 45.62(38.38 to 58.35) | 27.66(21.67 to 36.21) | 48.39(40.53 to 62.88) |
| Jamaica | 55.08(43.16 to 76.12) | 46.19(34.47 to 65.93) | 60.4(45.57 to 88.26) | 56.9(44.29 to 79.84) | 46.58(33.88 to 67.1) | 62.84(47.5 to 91.34) |
| Japan | 9.6(6.05 to 13.95) | 9.39(3.95 to 15.14) | 9.19(5.78 to 13.57) | 8.18(4.16 to 12.56) | 5.97(0.29 to 11.73) | 7.63(4.12 to 11.91) |
| Jordan | 39.6(28.03 to 59.28) | 23.62(13.23 to 38.35) | 42.22(30.11 to 63.17) | 39.87(28.18 to 59.21) | 25.36(13.76 to 40.57) | 41.76(29.68 to 62.4) |
| Kazakhstan | 78.27(63.36 to 102.38) | 66.94(50.48 to 94.32) | 81.55(65.69 to 107.04) | 80.19(64.91 to 104.72) | 67.62(51.74 to 93.98) | 83.04(66.6 to 108.88) |
| Kenya | 23.91(15.39 to 36.97) | 10.53(6.44 to 14.87) | 33.73(28.59 to 41.06) | 32.32(22.85 to 48.05) | 16.05(11.25 to 21.22) | 42.04(35.78 to 50.59) |
| Kiribati | 53.63(41.25 to 75.39) | 46.68(36.52 to 64.01) | 64.46(50.15 to 90.35) | 56.86(44.52 to 78.64) | 47.41(36.97 to 64.55) | 69.54(53.77 to 98.77) |
| Kuwait | 41.64(28.5 to 66.04) | 35.89(26.12 to 53.51) | 40(28.91 to 59.04) | 45.44(32.31 to 68.64) | 38.46(28.05 to 56.79) | 43.8(32.19 to 64.14) |
| Kyrgyzstan | 52.17(41.14 to 69.76) | 47.99(31.9 to 68.19) | 51.08(38.1 to 67.26) | 52.58(40.52 to 69.44) | 49.21(32.43 to 70.64) | 50.85(36.97 to 67.48) |
| Lao People's Democratic Republic | 65.88(51.57 to 90.74) | 71.91(56.99 to 97.44) | 61.58(46.55 to 86.05) | 69.25(52.88 to 96.99) | 76.4(59.89 to 104.48) | 65.03(48.12 to 92.44) |
| Latvia | 64.61(52.17 to 81.82) | 57.75(44.12 to 79.05) | 64.73(52.3 to 83.9) | 70.05(56.46 to 87.97) | 60.88(46.77 to 83.69) | 70.06(56.76 to 90.05) |
| Lebanon | 60.15(47.38 to 82.54) | 55.95(45 to 75.83) | 61.69(48.17 to 85.3) | 62.51(49.47 to 85.51) | 58.8(46.82 to 80.92) | 63.78(49.52 to 88.94) |
| Lesotho | 40.46(29.97 to 56.25) | 44.3(32.37 to 61.86) | 34.35(23.4 to 49.65) | 48.28(34.04 to 67.77) | 55.04(39.52 to 77.69) | 42.01(28 to 61.27) |
| Liberia | 39.55(28.58 to 58.49) | 45.31(33.51 to 63.93) | 37.93(26.67 to 56.22) | 40.66(27.69 to 60.4) | 50.35(36.52 to 70.96) | 38.43(25.39 to 58.04) |
| Libya | 69.09(55.37 to 93.45) | 75.51(60.27 to 101.99) | 67.53(53.65 to 91.39) | 72.38(58.1 to 97.86) | 79.47(63.82 to 107.77) | 70.31(55.5 to 94.6) |
| Lithuania | 37.28(26.81 to 49.78) | 27.04(14.83 to 43.95) | 40.31(28.38 to 55.21) | 41.42(30.75 to 54.48) | 28.94(16.16 to 46.24) | 43.94(31.06 to 59.78) |
| Luxembourg | 222.73(187.54 to 288.57) | 197.16(166.04 to 253.46) | 227.37(187.07 to 302.63) | 230.36(195.45 to 297.7) | 201.42(169.28 to 258.85) | 231.67(192.05 to 305.26) |
| Madagascar | 23.88(10.36 to 41.79) | 27.7(17.56 to 40.43) | 25.24(14.45 to 38.48) | 31.74(17.17 to 48.91) | 34.69(23.11 to 50.32) | 33.25(21.54 to 47.86) |
| Malawi | 28.03(9.2 to 49.62) | 19.64(10.58 to 30.57) | 35.65(23.48 to 52.21) | 36.4(17.13 to 60.9) | 25.93(13.98 to 39.72) | 43.29(29.35 to 61.93) |
| Malaysia | 43.87(33.72 to 62.54) | 44.8(33.47 to 63.54) | 43.71(32.05 to 64.14) | 43.96(32.92 to 61.68) | 44.62(32.34 to 63.93) | 43.79(31.27 to 64.13) |
| Maldives | 46.7(34.17 to 66.41) | 43.46(31.71 to 61.68) | 50.02(36.56 to 72.2) | 50.66(36.96 to 70.49) | 49.18(35.09 to 69.81) | 54.71(39.64 to 78.56) |
| Mali | 48.12(29.66 to 72.14) | 58.16(45.17 to 78.33) | 42.44(29.62 to 60.31) | 52.98(33.61 to 78.47) | 65.25(50.22 to 88.48) | 46.23(32.45 to 65.83) |
| Malta | 29.45(20.99 to 44.36) | 33.56(22.14 to 51.41) | 28.14(18.1 to 44.53) | 27.64(18.7 to 42.17) | 32.36(20.69 to 50.24) | 26.5(16.22 to 42.19) |
| Marshall Islands | 40.27(30.5 to 59.22) | 49.33(37.84 to 70.41) | 32.79(23.33 to 49.04) | 43.57(33 to 64.58) | 52.18(39.6 to 74.02) | 36.93(26.85 to 54.96) |
| Mauritania | 37.66(25.25 to 52.94) | 58.07(44.38 to 77.8) | 26.41(14.87 to 40.69) | 41.67(26.18 to 60.71) | 64.24(46.81 to 86.23) | 28.9(14.03 to 46.06) |
| Mauritius | 77.92(61.54 to 111.01) | 75.01(56.84 to 108.87) | 77.17(58.39 to 111.87) | 79.33(62.42 to 111.89) | 75.22(56.23 to 108.84) | 79.78(61.24 to 115.08) |
| Mexico | 3.84(-1.28 to 9.83) | -4.08(-9.35 to 0.35) | 7.94(3.31 to 14.37) | 9.14(4.03 to 15.77) | 0.97(-3.89 to 5.64) | 13.37(8.79 to 20.53) |
| Micronesia (Federated States of) | 80.51(62.64 to 114.63) | 90.41(73.01 to 119.73) | 71.7(56.51 to 96.8) | 90.15(72.16 to 126.61) | 96.44(76.93 to 130.5) | 84.1(66.07 to 116.56) |
| Monaco | 75.81(62.84 to 97.31) | 71.32(58.87 to 92.22) | 75.37(61.87 to 98.78) | 79.21(66.05 to 101.89) | 76.43(63.29 to 98.08) | 78(64.11 to 101.92) |
| Mongolia | 61.78(19.61 to 132.46) | 54.9(43.37 to 68.69) | 57.5(46.25 to 73.28) | 67.63(22.96 to 145.95) | 55.04(41.59 to 70.2) | 58.44(46.53 to 74.8) |
| Montenegro | 42.51(32.42 to 59.66) | 47.26(35.97 to 65.3) | 40.45(28.55 to 58.65) | 47.26(36.47 to 65.25) | 50.09(37.63 to 69.23) | 45.47(32.59 to 65) |
| Morocco | 78.47(65.72 to 100.38) | 82.38(67.48 to 106.25) | 77.77(64.07 to 101.96) | 83.71(69.38 to 107.28) | 87.02(70.48 to 112.58) | 83.39(68.39 to 108.7) |
| Mozambique | 36.05(18.78 to 61.78) | 26.39(17.01 to 40.32) | 41.36(30.39 to 57.8) | 44.68(25.94 to 72.84) | 33.73(21.89 to 49.25) | 49.07(36.6 to 68.17) |
| Myanmar | 60.04(46.1 to 83.08) | 59.09(43.89 to 83.39) | 61.6(46.25 to 87.94) | 60.62(45.32 to 84.99) | 62.28(45.17 to 89.9) | 61.5(45.07 to 89.44) |
| Namibia | 22.18(13.96 to 33.99) | 20.83(11.51 to 34.03) | 22.63(12.42 to 35.61) | 27.97(16.92 to 42.68) | 27.76(15.21 to 44.11) | 28.24(16.03 to 44.01) |
| Nauru | 49.94(37.57 to 70.14) | 58.22(46.81 to 77.19) | 47.77(37.29 to 66.01) | 58.61(44.93 to 81.37) | 62.94(50.11 to 85.01) | 58.04(45.78 to 79.69) |
| Nepal | 69.82(57.99 to 90.21) | 70.77(56.96 to 92.72) | 68.75(56.94 to 87.25) | 79.46(66.38 to 101.59) | 82.77(67.63 to 107.78) | 76.28(62.41 to 98.08) |
| Netherlands | 26.7(16.8 to 39.43) | 15.32(3.13 to 29.55) | 29.65(17.2 to 45.69) | 27.54(16.78 to 40.82) | 16.15(4.12 to 31.62) | 30.22(16.9 to 47.05) |
| New Zealand | 64.22(51.2 to 85.13) | 58.32(44.15 to 79.64) | 62.74(47.49 to 84.62) | 62.47(49.63 to 81.31) | 53.08(40.08 to 72.05) | 62.14(48.15 to 82.84) |
| Nicaragua | 24.93(12.09 to 44.37) | 32.59(20.51 to 50.15) | 21.57(11.45 to 36.51) | 33.28(19.83 to 53.8) | 43.94(30.61 to 64.28) | 28.8(17.78 to 44.85) |
| Niger | 88.4(68.63 to 115.36) | 95.98(78.32 to 120.77) | 80.88(62.55 to 105.23) | 94.76(74.35 to 123.97) | 107.57(85.92 to 136.82) | 85.42(66.43 to 112.09) |
| Nigeria | 27.07(14.38 to 42.79) | 18.93(14.35 to 25.5) | 25.47(19.56 to 32.94) | 32.03(20.34 to 47) | 23.84(17.45 to 32.49) | 29.3(22.72 to 38) |
| Niue | 49.93(38.32 to 69.86) | 54.17(41.13 to 75.25) | 42.51(31.16 to 61.64) | 57.28(44.26 to 79.85) | 59.01(45.35 to 82.52) | 52.68(38.55 to 75.92) |
| North Macedonia | 63.84(50.53 to 87.97) | 60.2(44.83 to 86.4) | 65.43(50.69 to 92.22) | 69.28(54.88 to 94.34) | 63.34(47.23 to 89.89) | 71.53(55.93 to 100.09) |
| Northern Mariana Islands | 43.36(19.38 to 82.87) | 47.18(36.81 to 66.73) | 44.77(34.1 to 63.6) | 48.05(21.94 to 90.53) | 44.56(34.17 to 63.12) | 52.85(40.11 to 74.13) |
| Norway | 33.51(29.17 to 40.63) | 36.94(31.87 to 44.98) | 31.03(26.51 to 38.13) | 35.34(30.94 to 42.94) | 40.07(34.89 to 48.24) | 32.68(28.2 to 40.07) |
| Oman | 70.83(56.08 to 98.55) | 61.9(49.81 to 84.06) | 74.43(59.46 to 101.2) | 73.48(57.91 to 101.67) | 64.45(51.66 to 87.32) | 76.78(60.83 to 104.79) |
| Pakistan | 49.64(39.43 to 69.51) | 66.12(55.56 to 84.2) | 46.6(38.51 to 59.39) | 52.65(42.22 to 72.19) | 70.18(59.62 to 89.42) | 49.89(41.26 to 63.67) |
| Palau | 51.47(40.8 to 71.02) | 55.03(43.61 to 75.78) | 49.45(38.07 to 68.49) | 57.86(45.65 to 78.12) | 59(46.79 to 80.27) | 58.34(45.04 to 79.86) |
| Palestine | 70.39(53.8 to 102.15) | 67(50.26 to 96.58) | 70.78(53.18 to 103.56) | 71.59(53.69 to 103.72) | 68.88(52.2 to 97.72) | 72.19(54.45 to 105.86) |
| Panama | 32.02(22.09 to 48.12) | 32.29(19.96 to 52.24) | 32.18(21.19 to 51.85) | 37.43(26.68 to 54.87) | 37.29(23.96 to 58.84) | 38.07(26.37 to 58.37) |
| Papua New Guinea | 41.2(30.88 to 58.32) | 63.67(50.86 to 84.45) | 32.41(24.03 to 45.87) | 45.8(35.59 to 62.8) | 64.82(52.15 to 85.43) | 37.81(28.42 to 52.08) |
| Paraguay | 55.68(42.03 to 76.31) | 39.64(28.85 to 59.11) | 61.56(46.56 to 85.03) | 63.72(48.14 to 86.24) | 43.74(32.03 to 63.45) | 71.07(54.62 to 96.49) |
| Peru | 50.49(36.82 to 69.98) | 49.69(36.5 to 68.23) | 50.98(36.79 to 71.34) | 55.47(41.1 to 76.02) | 55.6(40.68 to 76.24) | 55.41(40.42 to 76.89) |
| Philippines | 7.07(0.48 to 15.13) | 8.28(2.82 to 14.45) | 7.45(2.47 to 12.84) | 15.23(8.23 to 24.34) | 15.54(9.51 to 23.05) | 16.61(10.79 to 23.74) |
| Poland | 42.29(36.57 to 52.24) | 23.79(18.65 to 30.69) | 45.75(39.31 to 56.16) | 45.55(39.51 to 56.02) | 24.55(19.34 to 31.33) | 49.09(42.28 to 59.81) |
| Portugal | 49.71(38.02 to 69.79) | 46.97(31.92 to 73.88) | 50.84(37.42 to 72.2) | 49.35(37.27 to 69.36) | 46.17(30.59 to 72.67) | 49.78(36.48 to 70.59) |
| Puerto Rico | 27.31(18.5 to 42.36) | 21.69(12.31 to 36.35) | 29.63(18.98 to 46.54) | 30.37(21.18 to 46.68) | 22.8(12.65 to 38.28) | 33.57(22.93 to 51.96) |
| Qatar | 47.31(31.36 to 77.34) | 56.14(39.74 to 85.21) | 45.26(31.64 to 68.54) | 49.85(34.77 to 77.14) | 60.26(42.59 to 90.88) | 45.64(31.76 to 69.07) |
| Republic of Korea | 38.99(29.02 to 54.29) | 33.67(22.5 to 49.67) | 39.68(28.42 to 56.78) | 38.63(29.02 to 53.85) | 32.18(20.82 to 48.21) | 38.61(27.9 to 54.68) |
| Republic of Moldova | 33.65(21.17 to 49.1) | 25.52(12.97 to 42.5) | 35.54(22.83 to 52.02) | 36.54(22.54 to 52.83) | 27.16(14.2 to 44.18) | 38.37(25.07 to 55.65) |
| Romania | 51.21(39.09 to 66.71) | 45.08(32.68 to 65.74) | 52.19(38.8 to 70.22) | 54.04(41.25 to 71.49) | 44.6(31.65 to 65.3) | 55.49(41.54 to 74.3) |
| Russian Federation | 40.53(36.14 to 46.93) | 35.23(31.35 to 41.03) | 38.59(34.28 to 44.75) | 40.82(36.53 to 47.27) | 34.2(30.39 to 40.22) | 38.92(34.59 to 44.97) |
| Rwanda | 25.51(13.22 to 38.83) | 25.89(14.93 to 39.08) | 25.49(14.37 to 39.09) | 31.01(17.71 to 47.64) | 31.64(17.23 to 48) | 30.41(16.86 to 45.76) |
| Saint Kitts and Nevis | 24.49(14.21 to 42.19) | 13.61(4.7 to 24.23) | 34.85(24.81 to 51.04) | 31.58(18.25 to 54.96) | 20.1(10 to 33.83) | 40.76(29.78 to 59.41) |
| Saint Lucia | 18.53(10.67 to 32.09) | 16.69(9.46 to 29.15) | 19.15(10.97 to 30.95) | 21.67(12.56 to 37.12) | 18.68(10.9 to 31.7) | 23.6(14.6 to 37.31) |
| Saint Vincent and the Grenadines | 23.79(13.19 to 39.5) | 21.95(13.52 to 35.12) | 22.45(12.29 to 36.26) | 30.11(19.22 to 47.92) | 25.7(16.42 to 40.75) | 29.93(18.5 to 45.64) |
| Samoa | 43.73(31.59 to 65.3) | 57.58(45.77 to 77.5) | 34.72(25.9 to 48.48) | 47.89(34.18 to 72.36) | 60.33(47.49 to 81.71) | 40.53(30.38 to 56.28) |
| San Marino | 60.93(49.43 to 80.15) | 57.4(45.76 to 75.43) | 60.61(49.45 to 79.44) | 63.62(51.72 to 83.37) | 58.51(45.58 to 77.25) | 62.71(49.96 to 81.24) |
| Sao Tome and Principe | 37.82(24.87 to 54.46) | 36.49(25.27 to 51.06) | 35.11(21.84 to 53.49) | 44.15(27.65 to 64.24) | 40.46(26.02 to 58.46) | 44.15(27.04 to 67.52) |
| Saudi Arabia | 50.08(38.66 to 71.46) | 57.64(46.27 to 80.05) | 47.36(36.38 to 68.07) | 50.88(38.36 to 72.5) | 60.32(47.72 to 83.42) | 47.44(35.39 to 68.89) |
| Senegal | 31.82(20.72 to 47.17) | 40(29.9 to 56.44) | 29.28(17.98 to 44.39) | 33.4(21.13 to 50.26) | 46.55(34.56 to 66.72) | 29.85(16.84 to 47.54) |
| Serbia | 49.94(38.22 to 71.11) | 39.35(26.68 to 56.97) | 52.91(38.81 to 77.08) | 52.66(39.96 to 75.04) | 39.39(25.67 to 57.91) | 56.11(40.92 to 80.91) |
| Seychelles | 92(74.16 to 129) | 103.6(81.81 to 142.7) | 84.47(65.62 to 118.57) | 100.32(81.85 to 137.11) | 116.93(91.47 to 159.77) | 91.86(71.61 to 126.71) |
| Sierra Leone | 58.26(35.17 to 93.45) | 83.51(66.38 to 106.8) | 48.39(33.47 to 66.96) | 62.32(36.41 to 97.42) | 91.97(73.32 to 119.81) | 51.69(35.68 to 71.71) |
| Singapore | 5.88(-2.36 to 15.18) | -6.09(-14.96 to 3.15) | 11.03(1.38 to 22.28) | 4.54(-3.67 to 13.83) | -8.57(-17.75 to 0.94) | 9.45(-0.02 to 20.66) |
| Slovakia | 33.68(22.8 to 50.7) | 30.79(18.63 to 46.91) | 34.48(22.1 to 52.66) | 37.58(26.16 to 55.14) | 32.52(20.3 to 49.43) | 39(26.28 to 57.87) |
| Slovenia | 23.42(12.58 to 38.34) | 10.08(-0.48 to 22.67) | 25.16(13.46 to 40.84) | 28.52(17.27 to 43.48) | 12.77(1.72 to 26.49) | 29.66(17.02 to 46.48) |
| Solomon Islands | 76.79(58.36 to 107.87) | 80.25(65.78 to 104.17) | 83.35(66.27 to 112.41) | 79.78(62.33 to 109.56) | 80.37(66.16 to 104.76) | 86.23(68.6 to 115.79) |
| Somalia | 26.54(14.54 to 39.43) | 26.18(16.64 to 38.37) | 30.97(19.41 to 45.58) | 32.77(20.87 to 46.52) | 31.95(20.21 to 46.52) | 36.96(24.34 to 53.23) |
| South Africa | 37.73(31.44 to 47.48) | 40.77(32.78 to 52.46) | 35.64(29.24 to 46.02) | 55.55(47.09 to 69.75) | 65.85(51.32 to 84.72) | 50.55(41.83 to 64.45) |
| South Sudan | 29.71(18.04 to 44.5) | 18.67(9.08 to 29.45) | 29.35(18.31 to 43) | 36.57(24.22 to 51.69) | 25.25(14.14 to 39.02) | 36.9(23.95 to 53.13) |
| Spain | 26.08(16.31 to 38.9) | 12.64(2.56 to 26.92) | 27.56(16.57 to 42.37) | 29.52(19.18 to 43.15) | 11.17(0.85 to 24.4) | 31.1(19.88 to 46.78) |
| Sri Lanka | 71.32(56.11 to 100.38) | 78.45(60.06 to 112.05) | 70.19(53.01 to 100.63) | 79.77(63.63 to 110.41) | 87.6(67.43 to 123.2) | 79.56(60.98 to 115.01) |
| Sudan | 81.45(66.4 to 111.11) | 82.63(67.43 to 109.61) | 79.87(65.94 to 104.68) | 86.51(70.5 to 117.02) | 88.38(70.66 to 119.62) | 84.23(68.73 to 110.81) |
| Suriname | 48.1(37.96 to 66.1) | 50.81(39.95 to 71.83) | 46.12(34.9 to 65.78) | 52.6(41.87 to 71.28) | 53.24(41.06 to 75.18) | 51.84(39.99 to 73.26) |
| Sweden | 45.08(34.41 to 62.31) | 43.66(31.23 to 63.26) | 43.57(32.4 to 63.2) | 48.89(38.65 to 65.22) | 48.79(36.71 to 68.02) | 46.88(35.4 to 66.19) |
| Switzerland | 33.93(23.5 to 50.15) | 24.05(12.33 to 41.1) | 36.82(23.25 to 55.98) | 35.09(24.35 to 50.95) | 25.8(13.37 to 43.28) | 37.23(23.9 to 56.13) |
| Syrian Arab Republic | 54.45(43.07 to 73.73) | 56.12(43.81 to 78.56) | 53.84(41.11 to 75.95) | 57.11(43.98 to 77.85) | 59.98(45.22 to 83.69) | 56.21(41.5 to 79.47) |
| Taiwan (Province of China) | 37.84(29.66 to 52.11) | 24.22(16.35 to 36.43) | 43.9(32.7 to 63.03) | 39.42(30.18 to 53.78) | 22.67(15.06 to 34.58) | 47.09(34.85 to 66.82) |
| Tajikistan | 114.18(94.15 to 144.55) | 115.04(94.39 to 149.18) | 111.5(88.44 to 141.65) | 120.39(97.3 to 153.09) | 122.89(99 to 160.49) | 116.65(91.12 to 151.58) |
| Thailand | 50.31(37.43 to 72.16) | 51.05(35.59 to 73.78) | 48.7(34 to 73.44) | 51.68(37.85 to 74.94) | 51.34(35.62 to 74.49) | 50.88(35.62 to 75.67) |
| Timor-Leste | 89.28(73.5 to 119.04) | 94.33(76.54 to 123.99) | 83.98(68.1 to 112.55) | 95.76(77.26 to 130.17) | 99.66(78.85 to 133.12) | 91.51(71.37 to 125.19) |
| Togo | 40.45(25.13 to 61.23) | 45.57(34.03 to 60.52) | 41.55(29.02 to 57.31) | 42.07(25.78 to 62.78) | 52.01(38.38 to 69.89) | 43.31(28.35 to 61.4) |
| Tokelau | 51.25(40.72 to 69.45) | 51.63(41.2 to 71.69) | 51.78(40.47 to 73.22) | 56.37(43.97 to 77.95) | 56.17(44.04 to 78.26) | 59.3(45.37 to 84.16) |
| Tonga | 43.37(33.94 to 58.03) | 38.32(28.22 to 53.77) | 45.68(34.86 to 62.69) | 48.04(37.65 to 63.73) | 38.45(28.64 to 54.45) | 53.6(41.22 to 73.25) |
| Trinidad and Tobago | 19.82(8.89 to 36.53) | 13.74(3.68 to 26.07) | 22.39(12.5 to 37.89) | 19.21(7.17 to 36.36) | 13.06(1.26 to 26.83) | 22.21(10.93 to 38.27) |
| Tunisia | 60.46(48.14 to 82.95) | 70.22(56.65 to 92.86) | 60.32(47.78 to 82.09) | 63.33(49.84 to 86.52) | 74.28(59.3 to 98.69) | 63.45(49.97 to 86.56) |
| Turkey | 48.09(33.19 to 71.24) | 41.49(27.58 to 61.28) | 49.84(34.01 to 73.99) | 49.28(33.68 to 71.62) | 43.6(28.67 to 63.94) | 50.72(34.52 to 75.64) |
| Turkmenistan | 66.71(50.14 to 90.65) | 62.29(44.71 to 84.24) | 70.18(56.82 to 91.54) | 67.53(51.87 to 91.43) | 62.97(45.55 to 85.53) | 70.5(56.57 to 91.6) |
| Tuvalu | 59.97(48.58 to 79.28) | 67.58(54.59 to 89.43) | 49.21(37.54 to 67.27) | 64.13(51.9 to 83.81) | 71.58(57.64 to 96) | 55.64(42.96 to 75.72) |
| Uganda | 29.28(17.62 to 44.29) | 22.28(13.15 to 35.83) | 37.74(26.48 to 53.79) | 35.31(23.14 to 51.88) | 27.1(16.13 to 42.66) | 44.27(31.33 to 62.98) |
| Ukraine | 30.63(17.03 to 47.74) | 21.44(10.19 to 35.55) | 30.99(16.43 to 49.61) | 30.85(16.41 to 48.8) | 18.98(7.39 to 33.22) | 31.1(15.8 to 50.21) |
| United Arab Emirates | 38.78(23.46 to 60.62) | 34.6(24.5 to 53.71) | 40.36(29.25 to 58.89) | 40.07(23.29 to 63.41) | 34.74(22.45 to 57) | 40.35(28.02 to 60.26) |
| United Kingdom | 66.83(57.38 to 83.46) | 68.69(58.01 to 87.07) | 64.58(53.99 to 80.96) | 68.76(58.93 to 85.78) | 68.97(58.67 to 86.44) | 67.15(56.69 to 83.67) |
| United Republic of Tanzania | 52.93(37.64 to 73.66) | 48.38(35.29 to 65.08) | 53.44(39.04 to 72.41) | 64.77(49.04 to 88.83) | 59.49(44.4 to 80.53) | 66.23(50.38 to 87.75) |
| United States of America | 52.97(43.9 to 72.26) | 41.03(34.1 to 52.12) | 55.67(46.44 to 71.71) | 50.39(42.01 to 67.3) | 38.67(32.01 to 49.3) | 52.86(44.12 to 68.02) |
| United States Virgin Islands | 49.23(38.66 to 69.15) | 42.42(32.57 to 60.26) | 49.25(37.6 to 68.09) | 56.73(44.27 to 79.02) | 46.1(34.88 to 65.8) | 57.8(45.06 to 78.32) |
| Uruguay | 186.76(158.36 to 233.71) | 104.15(84.61 to 134.19) | 214.5(182.35 to 267.44) | 194.47(165.48 to 240.25) | 103.8(84.55 to 134.07) | 218.91(187.55 to 272.97) |
| Uzbekistan | 141.27(119.07 to 176.78) | 142.2(118.05 to 182.8) | 137.85(111.41 to 176.75) | 148.95(124.29 to 187.6) | 153.98(126.3 to 199.9) | 143.64(116.01 to 183.49) |
| Vanuatu | 52.61(38.92 to 74.86) | 70.2(56.23 to 93.46) | 45.53(34.29 to 63.93) | 57.89(44.37 to 80.31) | 70.98(56.14 to 95.28) | 52.56(39.99 to 73.97) |
| Venezuela (Bolivarian Republic of) | 25.15(15.95 to 39) | 18.93(7.81 to 33.59) | 28.05(17.07 to 46.8) | 30.05(19.74 to 44.58) | 22.9(11.4 to 38.11) | 33.22(21.31 to 52.26) |
| Viet Nam | 70.9(55.45 to 96.48) | 65.16(50.08 to 92.82) | 74.45(58.3 to 101.68) | 71.86(54.1 to 99.45) | 68.25(50.08 to 97.38) | 75.83(58.31 to 104.54) |
| Yemen | 61.95(47.46 to 82.62) | 66.99(54.38 to 85.87) | 55.95(43.79 to 74.36) | 64.14(50.04 to 85.08) | 69.61(55.13 to 90.39) | 57.59(44.51 to 77.09) |
| Zambia | 27.73(16.93 to 40.87) | 17.92(8.7 to 29.77) | 35.11(23.69 to 51.1) | 32.73(20.8 to 48.46) | 22.55(11.07 to 36.74) | 39.83(26.83 to 58.06) |
| Zimbabwe | 39.25(24.26 to 61.02) | 41.68(29.1 to 59.58) | 32.75(21.81 to 48.04) | 46(27.28 to 71.33) | 50.63(36.2 to 71.27) | 37.66(24.63 to 54.4) |

Abbreviations: FPG fasting plasma glucose; DALY disability-adjusted life year; UI uncertainty interval; ASDR age-standardized death rate.
